# O-acetylation of the serine-rich repeat glycoprotein GspB is coordinated with accessory Sec transport

**DOI:** 10.1371/journal.ppat.1006558

**Published:** 2017-08-21

**Authors:** Ravin Seepersaud, David Sychantha, Barbara A. Bensing, Anthony J. Clarke, Paul M. Sullam

**Affiliations:** 1 San Francisco Veteran Affairs Medical Center, and the University of California, San Francisco, San Francisco, CA, United States of America; 2 Department of Molecular and Cellular Biology, University of Guelph, Guelph, Ontario, Canada; Boston Children's Hospital, UNITED STATES

## Abstract

The serine-rich repeat (SRR) glycoproteins are a family of adhesins found in many Gram-positive bacteria. Expression of the SRR adhesins has been linked to virulence for a variety of infections, including streptococcal endocarditis. The SRR preproteins undergo intracellular glycosylation, followed by export via the accessory Sec (aSec) system. This specialized transporter is comprised of SecA2, SecY2 and three to five accessory Sec proteins (Asps) that are required for export. Although the post-translational modification and transport of the SRR adhesins have been viewed as distinct processes, we found that Asp2 of *Streptococcus gordonii* also has an important role in modifying the SRR adhesin GspB. Biochemical analysis and mass spectrometry indicate that Asp2 is an acetyltransferase that modifies *N*-acetylglucosamine (GlcNAc) moieties on the SRR domains of GspB. Targeted mutations of the predicted Asp2 catalytic domain had no effect on transport, but abolished acetylation. Acetylated forms of GspB were only detected when the protein was exported via the aSec system, but not when transport was abolished by *secA2* deletion. In addition, GspB variants rerouted to export via the canonical Sec pathway also lacked *O*-acetylation, demonstrating that this modification is specific to export via the aSec system. Streptococci expressing GspB lacking O-acetylated GlcNAc were significantly reduced in their ability bind to human platelets *in vitro*, an interaction that has been strongly linked to virulence in the setting of endocarditis. These results demonstrate that Asp2 is a bifunctional protein involved in both the post-translational modification and transport of SRR glycoproteins. In addition, these findings indicate that these processes are coordinated during the biogenesis of SRR glycoproteins, such that the adhesin is optimally modified for binding. This requirement for the coupling of modification and export may explain the co-evolution of the SRR glycoproteins with their specialized glycan modifying and export systems.

## Author summary

Bacteria express a variety of structures on their surfaces that promote interactions with the host. The serine-rich repeat (SRR) glycoproteins have emerged as an important group of surface proteins on many streptococci and staphylococci that mediate their attachment to a range of host tissues. The SRR glycoprotein GspB of *Streptococcus gordonii* is a key virulence factor for developing infective endocarditis (an infection of heart valves), due to its ability to mediate bacterial binding to human platelets. Like all SRR adhesins, GspB is extensively glycosylated in the cytoplasm and is then exported to the cell surface via a specialized transporter, the accessory Sec (aSec) system. The reason why a dedicated transport system is necessary for export has remained elusive. Here we show that in addition to its role in export, one aSec component (Asp2) is also an enzyme that can transfer an acetyl group to *N*-acetylglucosamine residues on GspB during transport. Loss of GspB acetylation significantly impaired the ability of streptococci to bind human platelets, indicating that acetylation is essential for the binding activity of GspB. These findings add to our knowledge of novel protein glycosylation mechanisms utilized by bacteria, and highlight the importance of SRR glycan acetylation in bacterial-host interactions.

## Introduction

The serine rich repeat (SRR) glycoproteins are a large family of adhesins on the surface of many Gram-positive bacteria. Expression of the SRR adhesins has been directly correlated with colonization and the ability of these organisms to cause invasive disease [1] [2] [3] [4] [5] [6]. The biogenesis of these adhesins involves the intracellular O-linked glycosylation [7] [8] and transport of the glycoproteins to the bacterial surface by the accessory Sec (aSec) system [9] [10]. Glycosylation is initiated by a two-protein glycosyltransferase (Gtf) complex (GtfAB) that adds *N*-acetylglucosamine (GlcNAc) to serine and threonine residues within the SRR domains of the adhesins [7] [11]. Other Gtfs sequentially further modify the SRR domains by adding other glycan moieties to the GlcNAc core. The number and type of these Gtfs varies considerably between species, as do the resulting extent of glycan modification [12] [13] [14].

The aSec system is a dedicated transporter that exclusively mediates the transport of SRR glycoproteins [9] [15]. This system is comprised of SecA2 (the motor protein), SecY2 (the translocon channel) and three to five accessory Sec proteins (Asps) that are essential for SRR glycoprotein transport (reviewed in [16]). The exact role of the Asps in transport is uncertain. Our previous studies with the SRR adhesin GspB of *Streptococcus gordonii* have identified numerous protein-protein interactions between Asps1-3, which are located intracellularly. Disruption of some of these interactions abolishes aSec transport, indicating a coordinated role for the Asps in export [17]. Asp2 and Asp3 can bind GspB directly and Asp1-3 appear to enhance the binding of the GspB preprotein to membrane-associated SecA2 [18] [19]. These findings indicate that the Asps are essential for key stages of SRR protein translocation, either by acting upon the substrate or other members of the aSec system [16].

Although the post-translational modification and export of the SRR adhesins have been largely viewed as separate pathways, our previous studies indicate that at least Asp2 may have a role in both processes. Modeling of the predicted structure of Asp2 suggests that it shares similarities with numerous glycan-modifying enzymes [20]. Moreover, a point mutation within a predicted catalytic triad of Asp2 resulted in an altered glycoform of GspB, which was exported freely by the aSec system, but had reduced binding to its platelet-localized ligand, sialyl-T antigen. These findings suggested that in addition to the known Gtfs, Asp2 may also modify the glycan on GspB [20]. However, the precise role of Asp2 in the post-translation modification of the SRR adhesins was unknown. Here, we show that Asp2 mediates the *O*-acetylation of GlcNAc residues on GspB. Furthermore, this modification is transport dependent, occurring exclusively when the substrate is transported through the aSec pathway. These results indicate that Asp2 serves as a nexus for the two major processes in SRR adhesin biogenesis, through its role in both transport and glycan modification. The finding that the acetylation of GspB only occurs during aSec transport indicates that glycan modification and transport are tightly linked processes and explains at least in part the need for a dedicated export system.

## Results

### Asp2 modifies GlcNAc residues on GspB

**Fig 1:**
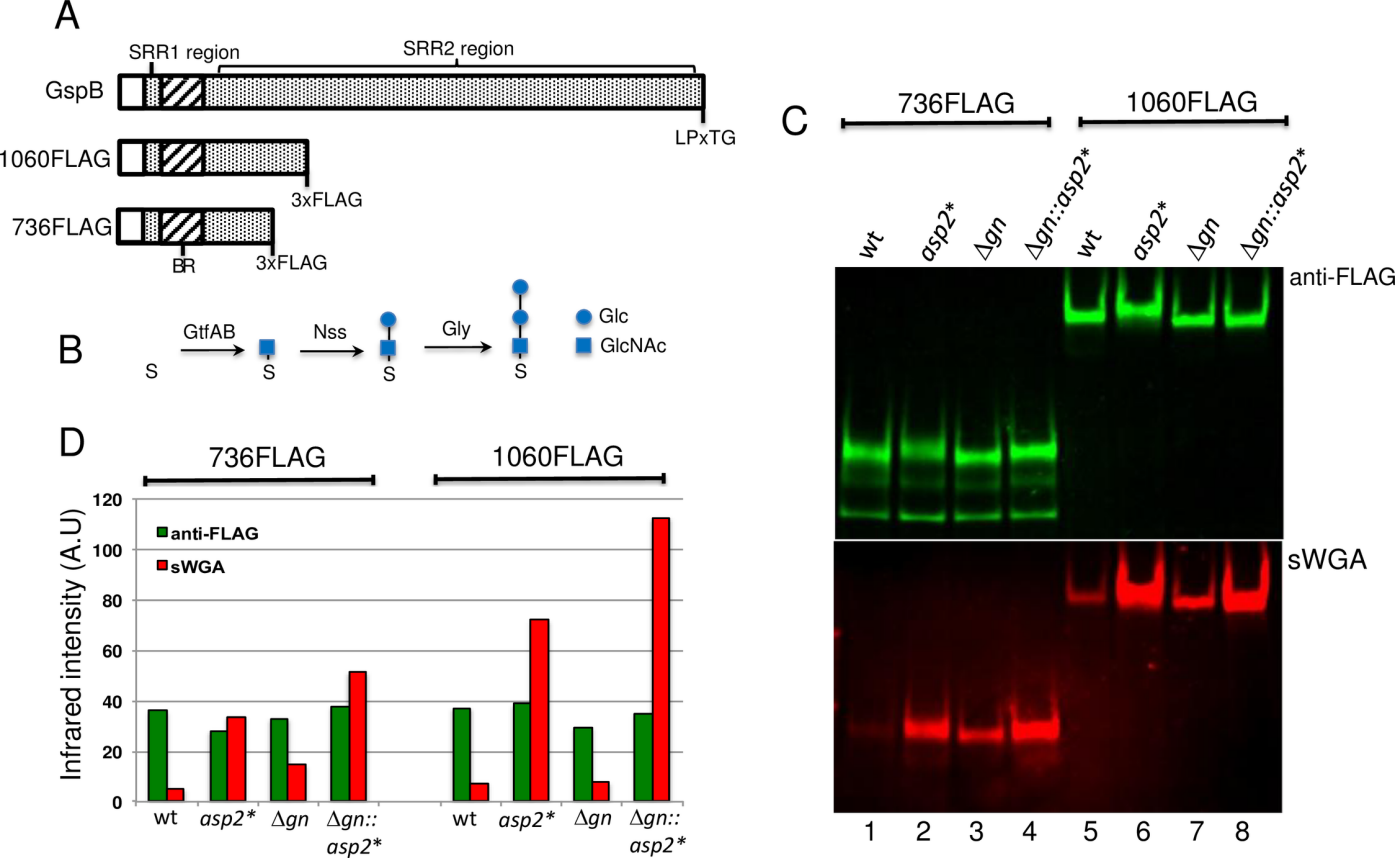
Reactivity of GlcNAc-modified GspB with sWGA. A) Schematic representation of wild-type GspB, GspB1060flag and GspB736flag. The first and second glycosylated serine rich repeat regions (SRR1 and SRR2) within GspB are shown together with the non-glycosylated substrate binding-region (BR). B) Schematic representation of GspB glycosylation by GtfAB, Nss and Gly. Glycan structures obtained from MALDI-TOF profiling of SRR1 glycosylated by the designated Gtf (S1 Fig and S2 Fig) are shown. C) Western blot analysis of GspB736flag and GspB1060flag export by parental strains PS1225 (lane 1) and PS921 (lane 5), their *nss* and *gly* deletion variants (Δ*gn*) PS3309 (lane 3) and PS3318 (lane 7) and their corresponding derivative strains harboring the S362A mutation within asp2 (asp2*), PS3539 (lane 2), PS3540 (lane 4), PS3541 (lane 6), PS3542 (lane 8). Culture media was collected from exponentially growing strains and proteins were separated by SDS-PAGE and subjected to Western blot analysis using anti-FLAG antibodies and biotinylated sWGA to determine GspB levels and GlcNAc reactivity, respectively. D) Densitometry analysis of GspB736flag and GspB1060flag levels and GlcNAc reactivity. The y-axis represents GspB levels and GlcNAc reactivity based on band intensity analysis via LI-COR imaging. Western and lectin blot analysis of secreted GspB variants, together with corresponding densitometry analysis, are representative results from 3 different genetic transformants.

We have shown previously that the site-specific replacement of residues that comprise a predicted Ser362-Asp452-His482 catalytic triad within Asp2 altered the reactivity of GspB with the GlcNAc-specific lectin sWGA, indicating a change in the glycan decorating the adhesin [20]. However, it remained unclear as to precisely how the glycan had been altered, as a consequence of the Asp2 mutations. The GtfAB complex mediates the O-linked transfer of GlcNAc to the SRR regions of GspB [7] [8]. Two additional Gtfs (Nss and Gly) sequentially add glucose residues to this glycan moiety (Fig 1B, S1 Fig and S2 Fig) (consistent with glycosylation of the SRR adhesin Fap1 [14]). Based on these findings, we first examined which components of the glycan were affected by an Asp2^S362A^ mutation. To assess changes in glycosylation, we used two variants of GspB (GspB736flag and GspB1060flag) (Fig 1A), containing C-terminal truncations and a 3xFLAG tag. These GspB variants lack cell wall anchoring domains, and are thus secreted into the culture media by *S*. *gordonii* strain M99, thereby facilitating the analysis of transport activity.

As compared with GspB variants made in a WT background, both GspB736flag and GspB1060flag showed a marked increase in sWGA reactivity, when exported by the Asp2^S362A^ expressing M99 variant, (Fig 1C, lane 2 versus 1 and lane 6 versus 5). Higher sWGA reactivity was seen in the longer GspB forms (Fig 1C and Fig 1D) suggesting the glycan change resulting from the Asp2^S362A^ mutation is present throughout the SRR2 region. The amounts of GspB exported were comparable among WT and Asp2 mutant strains as determined by anti-FLAG reactivity (Fig 1C and 1D), indicating that the changes in sWGA reactivity seen in GspB as a consequence of the Asp2^S362A^ mutation were not due to increased GspB production, but instead, resulted from differences in glycan composition.

To better define the glycan modified by Asp2, the Asp2^S362A^ mutation was introduced to a variant of M99, in which *gly* and *nss* had been deleted. Loss of *gly* and *nss* results in GspB variants glycosylated only by GtfAB, which is known to deposit GlcNAc only (Fig 1B, [7] [11]). Mutagenesis of *asp2* in this variant also resulted in forms of GspB736flag and GspB1060flag with increased sWGA binding (Fig 1C, lanes 4 versus 3 and lanes 8 versus 7). In contrast, no sWGA reactivity was observed with the GspB variants expressed by *gtfA*-deletion strains (S3 Fig), demonstrating that Asp2 modifies GlcNAc exclusively.

### Saponification of GspB reproduces the Asp2^S362A^ phenotype

We previously found via predictive modeling that the putative catalytic triad of Asp2 was conserved in numerous esterases [20]. However, O-linked GlcNAc typically does not have any ester-linkages. As some esterases and acyltransferases share a common fold and have similar mechanisms of action [21], we explored whether Asp2 functions as an acyltransferase that modifies GlcNAc residues. Since ester linkages to carbohydrates are base-sensitive [22], we assessed whether mild-base ester hydrolysis (saponification) altered the sWGA reactivity of GspB. Culture media containing secreted GspB736flag or GspB1060flag were treated with 100 mM NaOH and then probed with sWGA.

**Fig 2:**
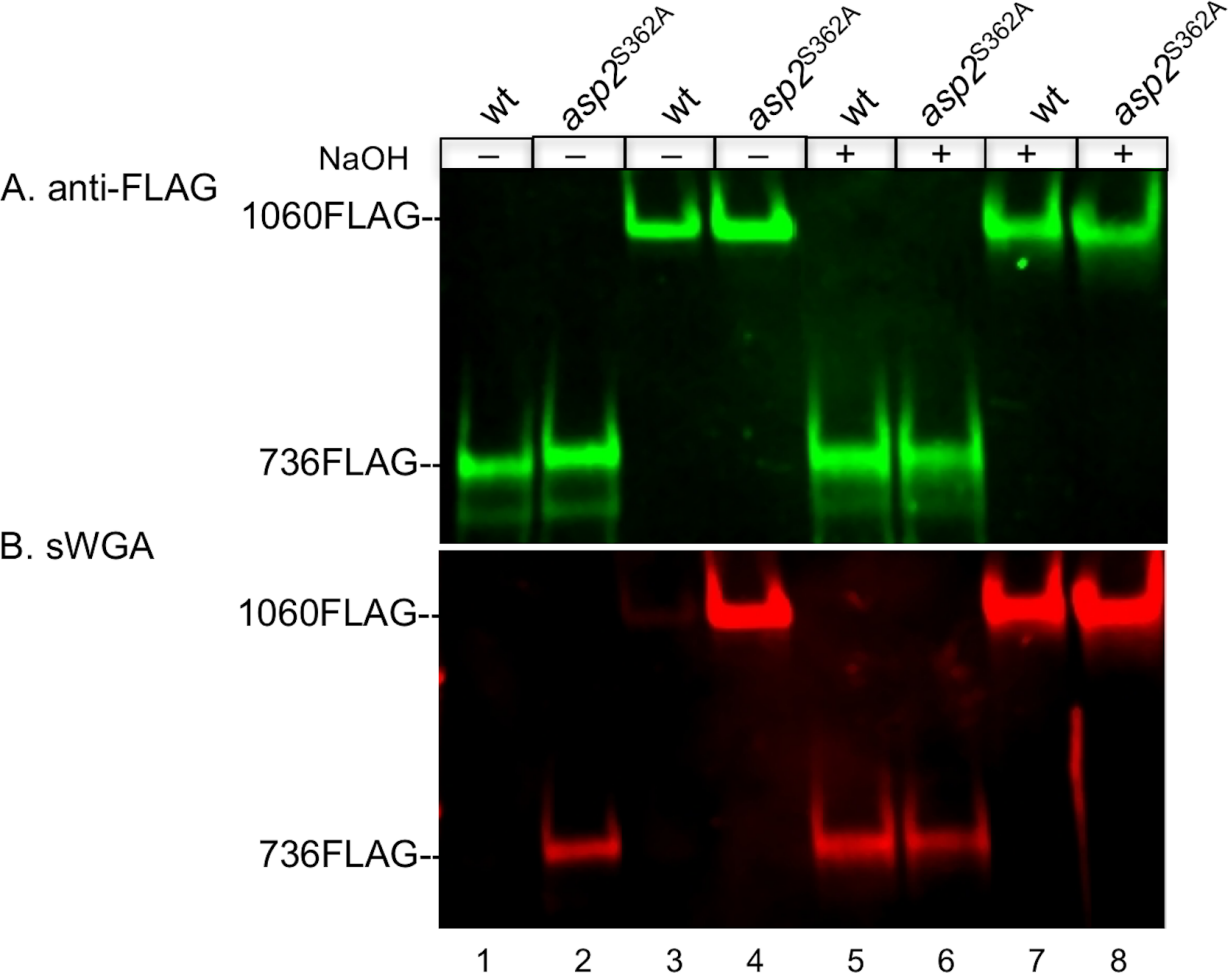
Saponification analysis of exported GspB736flag and GspB1060flag. A) GspB736flag and GspB1060flag secreted from parental strains PS1225 (lane 1) and PS921 (lane 3) and the corresponding derivative strains harboring the S362A mutation within *asp2*, PS3539 (lane 2), PS3540 (lane 4) were separated by SDS-PAGE and subjected to Western blot analysis using anti-FLAG antibodies to detect GspB levels. Saponified reaction products were obtained through incubation with 100 mM NaOH to release ester-linked acetate (lanes 5–8). B) Blot A was simultaneously probed for GlcNAc reactivity of GspB736flag and GspB1060flag before and after saponfication. GlcNAc reactivity was assessed by lectin blot analysis using biotinylated sWGA as a GlcNAc probe. Western and lectin blot analysis of secreted GspB variants are representative of 3 different genetic transformants analyzed from each strain.

In control studies, mild-base treatment did not alter the overall amounts of either GspB variant (Fig 2A) demonstrating that the conditions used for saponification did not degrade GspB. However, saponification did significantly increase the sWGA reactivity of secreted wild-type GspB736flag and GspB1060flag (Fig 2B, lanes 1 versus 5 and lanes 3 versus 7). In contrast, the sWGA reactivity of the same two variants exported from an Asp2^S362A^ background was unchanged by this treatment (Fig 2B, lanes 2 versus 6 and lanes 4 versus 8). Moreover, the increase of sWGA reactivity following mild base treatment of both GspB variants produced in a WT Asp2 background was comparable to the sWGA reactivity seen from their untreated catalytic mutant counterparts (Fig 2B, lanes 2 versus 5 and lanes 4 versus 7). These findings demonstrate that the increase in sWGA reactivity seen in Asp2^S362A^ mutant strains is due to a loss of an ester-linked chemical group on GlcNAc residues within the glycan of GspB, and suggest that Asp2 is a GlcNAc modifying enzyme.

### Asp2 O*-*acetylates GlcNAc moieties on GspB

**Fig 3:**
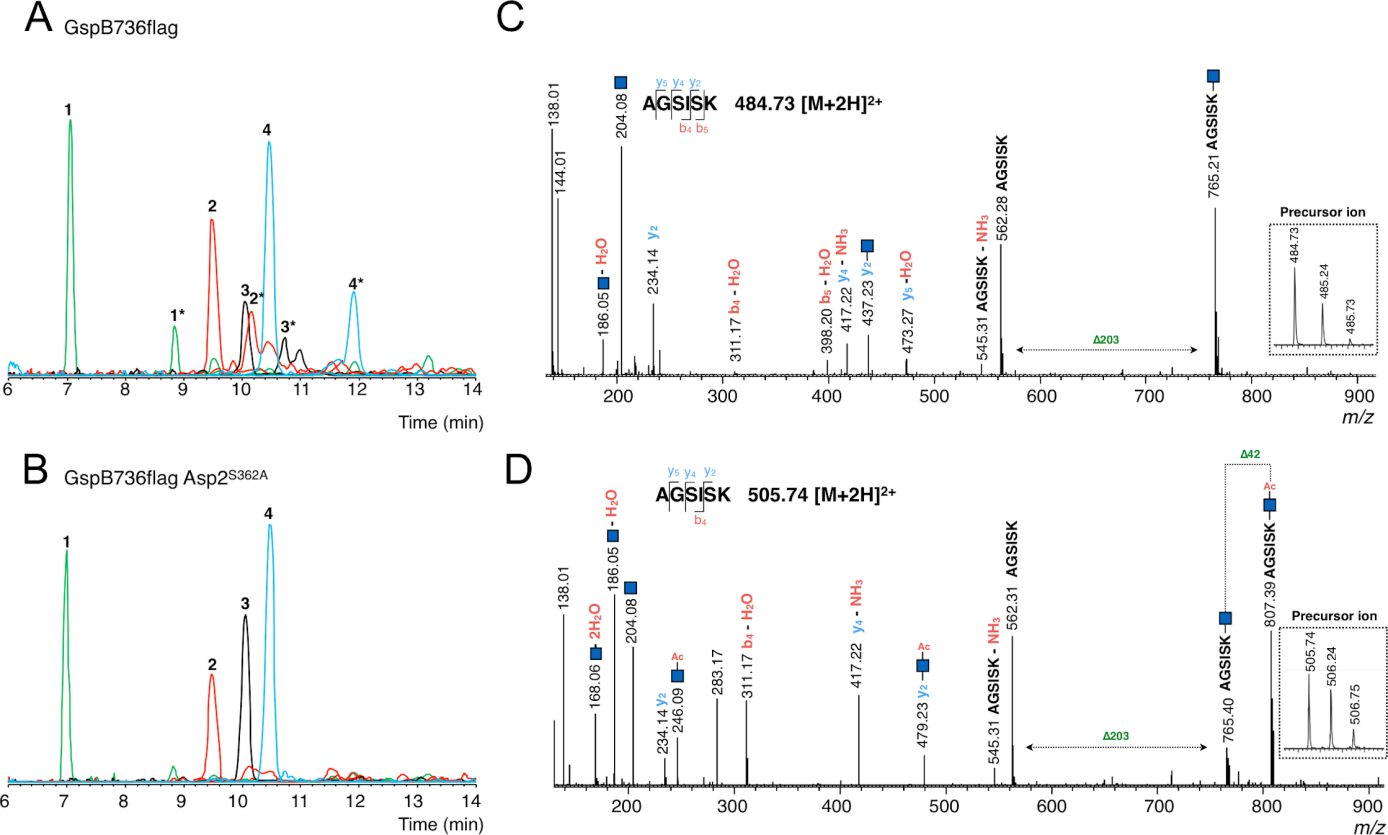
Q-TOF LC/MS analysis of GspB736flag after trypsin, Lys-C, and Glu-C protease digestion. Extracted ion chromatograms for all detected glycopeptides derived from GspB736flag secreted from A) a Δ*gly-nss* strain (PS3309) or B) a Δ*gly-nss* Asp2^S362A^ strain (PS3540). Peaks with the same peptide backbone are designated with the same color (green, red, black and blue, respectively) and asterisks denote those containing an O-acetylated glycopeptide. C) Fragmentation spectrum of peak 1, *m/z* = 484.73 [M+2H]^2+^ O-glycosylated at two positions. D) Fragmentation spectrum of peak 1*, *m/z* = 505.74 [M+2H]^2+^ O-glycosylated at two positions and O-acetylated at one position. *O*-GlcNAc modifications are designated by a blue square. MS spectra of precursor ions are shown in the insets.

To examine the influence of Asp2 activity on glycosylation, we subjected the Δ*gly-nss* glycoform of GspB736flag from both Asp2 and Asp2^S362A^ backgrounds to Q-TOF LC/MS analysis with collision-induced dissociation (CID) fragmentation. Because glycosylation of GspB occurs in the highly repetitive SRR2 region, which lacks Lys and Arg residues, we digested GspB736flag with trypsin, Lys-C, and Glu-C proteases simultaneously, to achieve better peptide coverage. To detect glycopeptides within the digests, we searched MS/MS scans for the production of diagnostic GlcNAc oxonium ions (*m/z* 204.08) that were generated from broken O-glycosidic linkages. Four major precursor ions were found in the extracted ion chromatogram (peaks 1 to 4) of the GspB736flagΔ*gly-nss* glycopeptides (Fig 3A). MS/MS analyses of these ions displayed characteristic neutral loss of GlcNAc (*m/z =* 203.08) and provided modest coverage of *y-* and *b-* type ions within a tolerance of 0.02 (Fig 3C, S4A, S5A and S6A Figs and S1 Table). These glycopeptides were mapped to the SRR2 region of GspB and the number of GlcNAc modifications could be determined (Table 1 and S1 Table). However, this method did not allow for the precise mapping of the *O*-GlcNAc sites within the glycopeptides.

**Table 1:** List of GspB736flag glycopeptides identified by Q-TOF LC/MS.

Peak	RT	*m*/z	# GlcNAc	# *O-*AcGlcNAc	Peptide
1	7.013	968.46	2	0	^**602**^AGSISK^**607**^
1*	8.848	1010.48	1	1	^**602**^AGSISK^**607**^
2	9.473	2317.93	6	0	^**660**^SASTSSSVSASE^**671**^
3	10.048	2301.94	6	0	^**672**^SASTSASVSASE^**683**^
2*	10.109	2359.96	5	1	^**660**^SASTSSSVSASE^**671**^
4	10.435	1762.81	4	0	^**624**^SASASLVTSK^**633**^
3*	10.73	2343.92	5	1	^**672**^SASTSASVSASE^**683**^
4*	11.882	1804.82	3	1	^**624**^SASASLVTSK^**633**^

RT, retention time.

* Glycopeptide containing *O-*AcGlcNAc.

Fractions containing the four SRR2-derived glycopeptides were found to elute from the reverse-phase column a second time with respective longer retention times (peaks 1* to 4* of Fig 3A). Each of the additional precursor ions detected in these fractions were 42.01 Da larger in mass compared to their corresponding partner glycopeptides, indicating the presence of an acetyl group. Their MS/MS spectra displayed neutral loss of 245.09 Da, and fragment ions at *m/z* 246.09 were also detected, which is consistent with an *O*-acetyl GlcNAc oxonium ion (Fig 3D, S4B, S5B and S6B Figs and S1 Table). Although the exact position of the O-acetylated GlcNAc residue within the glycopeptide could not be determined by MS/MS, some of the *O*-acetylated glycopeptides formed two peaks in the extracted ion chromatogram, suggesting that alternate sites of addition change peptide retention time. These findings directly demonstrate that SRR2 glycopeptides of GspB736flag from a Δ*gly-nss* background contain variable subpopulations of unmodified and O-acetylated GlcNAc residues.

The same glycopeptides were detected in GspB736flag from the Δ*gly-nss asp2*^S362A^ background and the extracted ion chromatogram indicated that their degree of glycosylation was not affected (Fig 3). However, whereas the LC chromatogram suggested the presence of a peak corresponding to peak 1* of Fig 3A, the extracted ion chromatogram was completely devoid of any ions corresponding to *O*-acetylated glycopeptides (S7 Fig). Of note, saponification of the above four acetylated glycopeptides of GspB736flag resulted in an extracted ion chromatogram identical to that seen of GspB736flag from an Asp2^S362A^ background (S8 Fig). These data indicate that Asp2 is responsible for the *O*-acetylation of GlcNAc residues within the SRR2 region of GspB and that GlcNAc *O*-acetylation results in the decrease in sWGA binding to secreted GspB observed above.

### Asp2 exhibits acetylesterase activity *in vitro*

**Fig 4:**
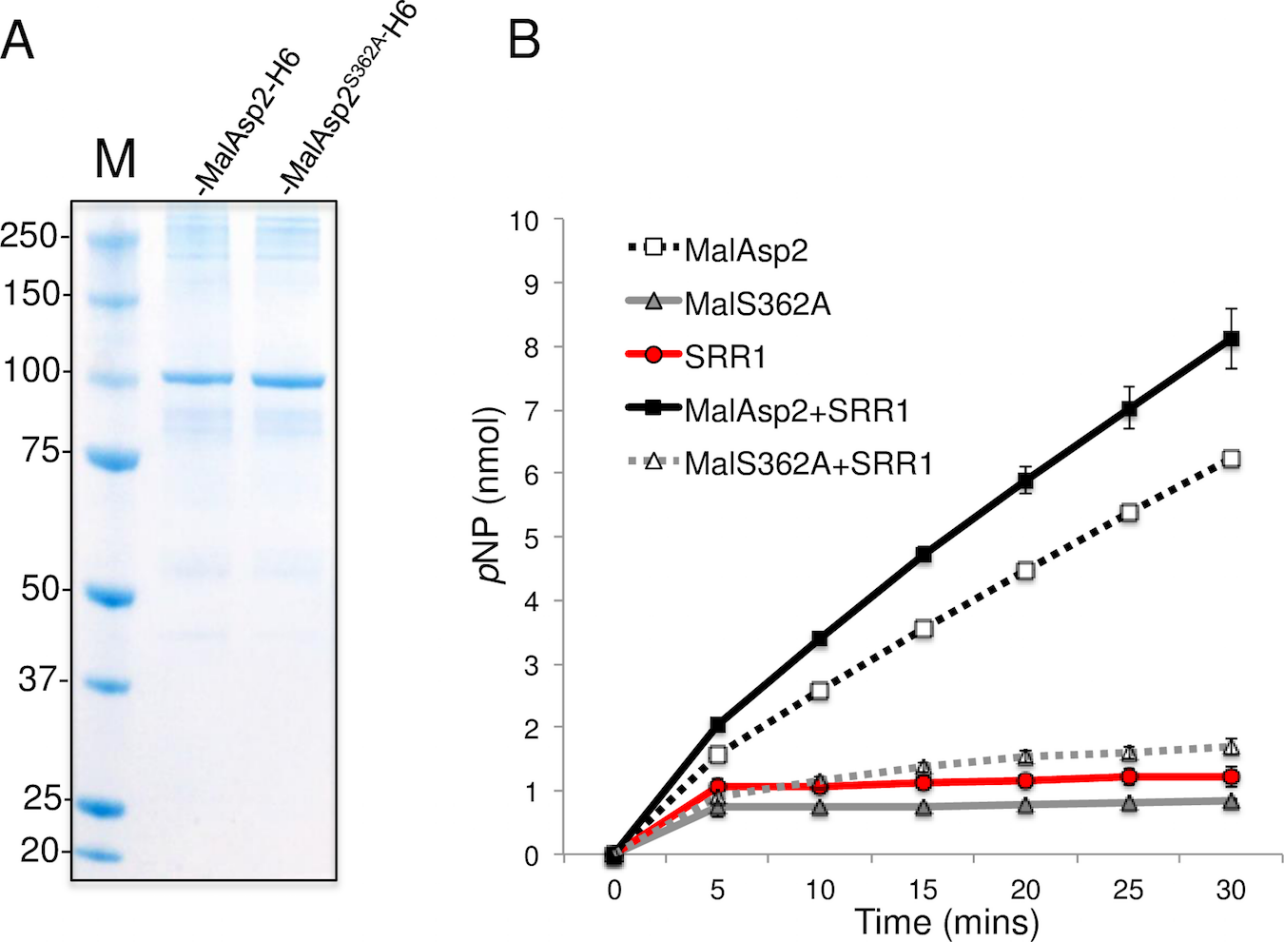
Determination of acetylesterase activity of Asp2. A) MalE-Asp2-H6 and the MalE-Asp2^S362A^-H6 catalytic mutant following expression in *E*. *coli* BL21 were purified on amylose resin, followed by Ni-NTA purification, and were analyzed by SDS-PAGE and Coomassie staining of 5 μg of the final purified recombinant protein. B) Time course of *p*-nitrophenol release from *p*NP-Ac in the presence of glycosylated SRR1 (red circle), MalE-Asp2-H6 (black open square), MalE-Asp2^S362A^-H6 (grey triangle), SRR1 with MalE-Asp2-H6 (black square) or SRR1 with MalE-Asp2^S362A^-H6 (grey open triangle). Reactions were incubated at 25°C in 50 mM sodium phosphate buffer (pH 7) and rates of *p*-nitrophenol release were measured spectrophotometrically at 405 nm. Assays were performed in triplicate and data expressed as mean ± S.D nmol *p*NP released. Results shown are representative of at least two independent experiments.

To directly assess the enzymatic activity of Asp2, we examined the ability of the recombinant protein to hydrolyze the acetyl donor *p*-nitrophenyl acetate (*p*NP-Ac). Of note, many acetyltransferases function as weak esterases in the absence of acceptor substrates when assayed *in vitro* as water will serve as the acceptor ligand for the acetyl group resulting in the release of acetate [23]. Using a MalE-Asp2H6 fusion protein and its predicted catalytic mutant, MalE-Asp2^S362A^-H6 (Fig 4A), hydrolysis of *p*NP-Ac was only seen with wild-type Asp2. This hydrolysis was increased in the presence of the glycosylated SRR1 domain of GspB (Fig 4B). MalE-Asp2^S363A^H6 only exhibited *p*NP-Ac hydrolysis levels on par with background levels and failed to further stimulate *p*NP-Ac hydrolysis following co-incubation with the SRR1 acceptor substrate. Western blot analysis of SRR1 after co-incubation with *p*NP-Ac and MalE-Asp2H6 revealed no change in sWGA reactivity, an indicator of GspB glycan *O*-acetylation (S9 Fig), suggesting that *in vitro*, the conditions used above do not entirely recapitulate the conditions *in vivo* for the transfer of O-acetyl groups to the glycan. Nonetheless, collectively these findings demonstrate that the Asp2 has *O*-acetylesterase activity and can hydrolyze acetyl donor substrates.

### Mutagenesis of the Asp2 catalytic site does not impair GspB export

**Fig 5:**
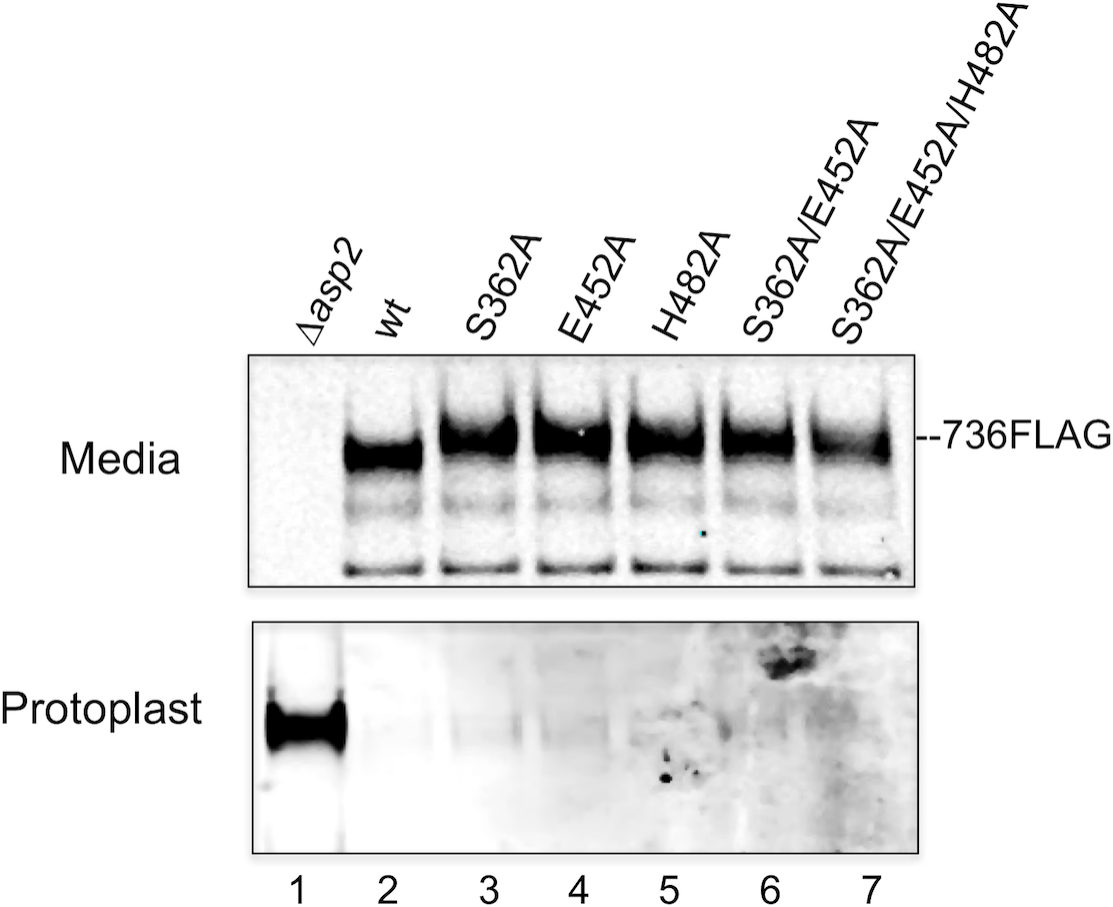
Effects of mutating the Asp2 catalytic triad upon the export of GspB736flag. Western blot analysis of Asp2-dependent export of GspB736flag by *S*. *gordonii* Δ*asp2* strain PS1244 (lane 1), parental strain PS1225 (lane 2) and derivative strains harboring the designated alanine substitution within the catalytic triad of Asp2, PS3539 (lane 3), PS3551 (lanes 4), PS3552 (lane 5), PS3553 (lane 6), PS3554 (lane 7). Culture media (M) and protoplasts (P) were collected from exponentially growing strains. Proteins were separated by SDS-PAGE and analyzed by Western blotting, using anti-FLAG antibody to detect GspB736flag. The data shown is representative of at least three different genetic transformants for each strain.

Asp2 is required for transport of GspB by the aSec system, with deletion of *asp2* abolishing GspB export and resulting in the retention of the SRR adhesin in the bacterial cytosol [9] [10] [17] [20]. To assess whether a loss Asp2 catalytic activity affects GspB transport, we compared the export of GspB736flag and GspB1060flag in a series of Asp2 catalytic mutants. As compared with the WT strain expressing Asp2, GspB736flag and GspB1060flag were comparably transported by isogenic variants expressing Asp2^S362A^, Asp2^E452A^ or Asp2^H482A^. The levels of the SRR adhesins detected in the culture supernatant were similar to those observed in the WT strains, while only trace amounts were detected in the protoplasts (Fig 5, S10 Fig, lane 2–5). To ensure that the enzymatic activity of Asp2 was entirely abolished, we also tested a variant containing an alanine replacement in all three putative catalytic residues. As was seen with the single amino acid substitutions, aberrantly glycosylated GspB was freely secreted by this variant, indicating that aSec transport was intact (Fig 5, S10 Fig, lane 6–7). Collectively, these findings show that the loss of Asp2 catalytic activity has no impact on aSec transport, and thus, the aberrant glycoform generated by Asp2 mutagenesis is not an artifact of altered transport. Instead, these results demonstrate that Asp2 is a bifunctional protein that directly acetylates GlcNAc moieties on GspB.

### Asp2 *O*-acetylates GlcNAc only during accessory Sec export

**Fig 6:**
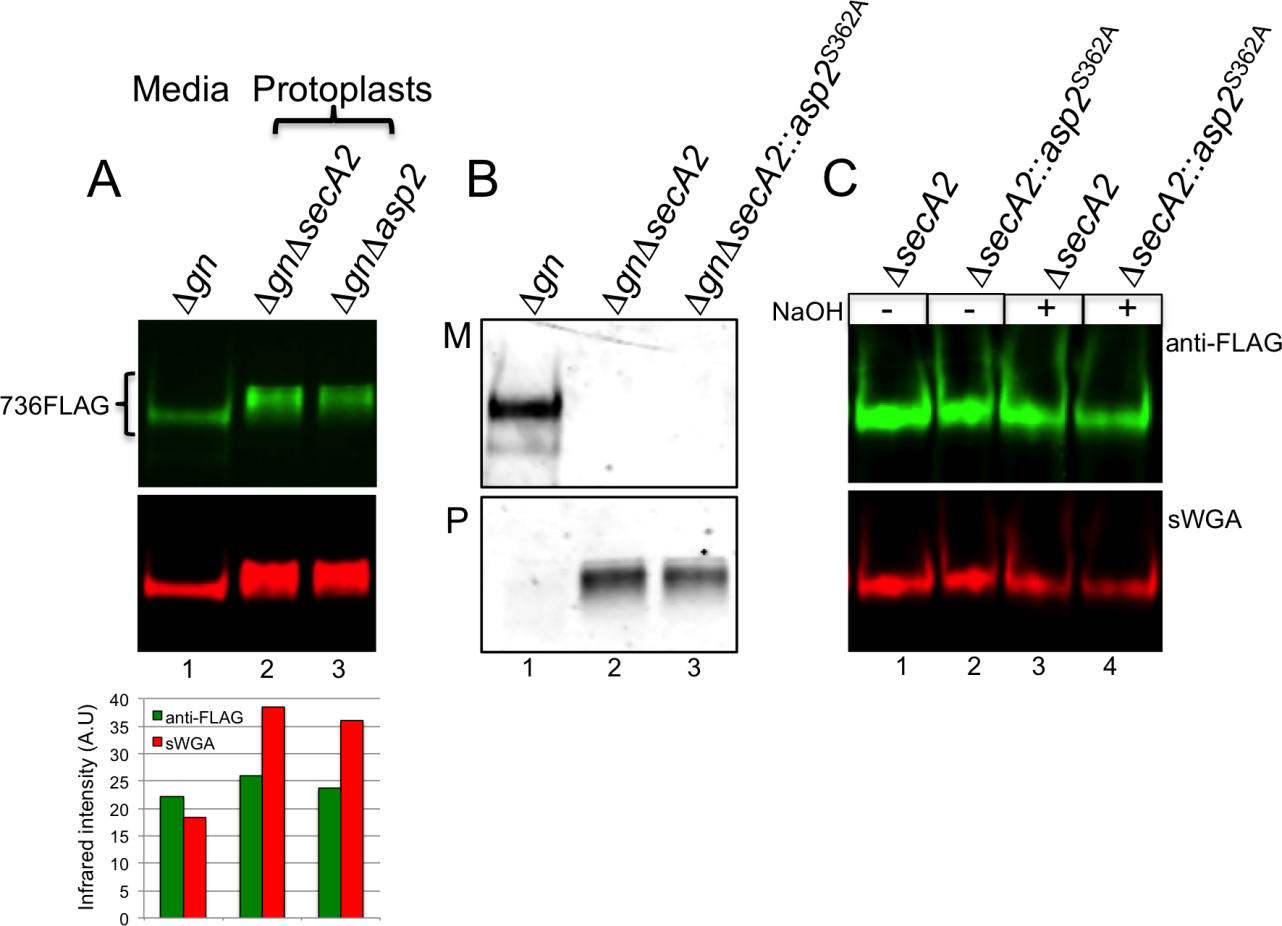
Effects of transport upon sWGA reactivity of GspB736flag. A) Upper panel shows Western blot analysis of GspB736flag exported from a *nss* and *gly* deletion (Δ*gn*) variant derivative strain PS3309 (lane 1) and intracellular GspB736flag expressed in the aSec deficient *nss* and *gly* deletion variant strains PS3310 (lane 2) and PS3584 (lane 3). Anti-FLAG antibodies were used to measure GspB736flag protein levels, while GlcNAc reactivity was assessed by lectin blot analysis using biotinylated sWGA as a GlcNAc probe. Lower panel shows densitometry analysis of GspB736flag levels and GlcNAc reactivity was determined by band intensity analysis via LI-COR imaging. B) Western blot analysis of GspB736flag exported from strains PS3309 (Δ*gn*)(lane 1), PS3310 (Δ*gn*Δ*secA2*)(lane 2) and PS3605 (Δ*gn*Δ*secA2*::*asp2*^S362A^)(lane 3). (M) media fraction, (P) protoplast fraction. GspB736flag was detected with anti-FLAG antibodies. C) GlcNAc reactivity of GspB736flag before and after saponfication of GspB736flag obtained from protoplasts in the defined aSec background. Non-exported GspB736flag was expressed in the following accessory Sec/Δ*gn* deletion strains, PS3310 (lane 1), PS3605 (lane 2). Saponified intracellular GspB736flag was achieved through incubation with 100 mM NaOH to release ester-linked acetate. Western and lectin blot analysis of secreted GspB variants, together with corresponding densitometry analysis, are representative of at least three different genetic transformants analyzed for each strain.

When export of GspB was abolished by an *asp2* or *secA2* deletion, the retained, intracellular glycoform of the adhesin differed in sWGA reactivity, as compared to the WT, secreted glycoform (Fig 6A). In particular, the intracellular glycoforms of GspB736flag showed a high level of sWGA reactivity, unlike the wild-type secreted form, and instead resembled the GspB glycoform secreted by the Asp2^S362A^ mutant. This indicated that the intracellular glycan of GspB lacked *O*-acetylated GlcNAc, suggesting that acetylation may be transport-dependent.

To further address this issue, we expressed GspB736flag in an isogenic variant of M99 (PS1226), in which aSec export was abolished because of a short, in-frame deletion within *secA2* [24]. As expected, the GspB variants were entirely retained in the cytosol (Fig 6B). Intracellular GspB736flag showed high levels of sWGA reactivity, which were unchanged following mild-base treatment (Fig 6C, lanes 3 & 4 versus lanes 1 and 2). These findings indicate that the GlcNAc moieties on intracellular GspB are not acetylated, suggesting that Asp2 does not modify GspB independently of transport, unlike GtfAB, Gly and Nss [25]. Instead, O-acetylation of GlcNAc by Asp2 appears to only occur concomitant with substrate transport.

**Fig 7:**
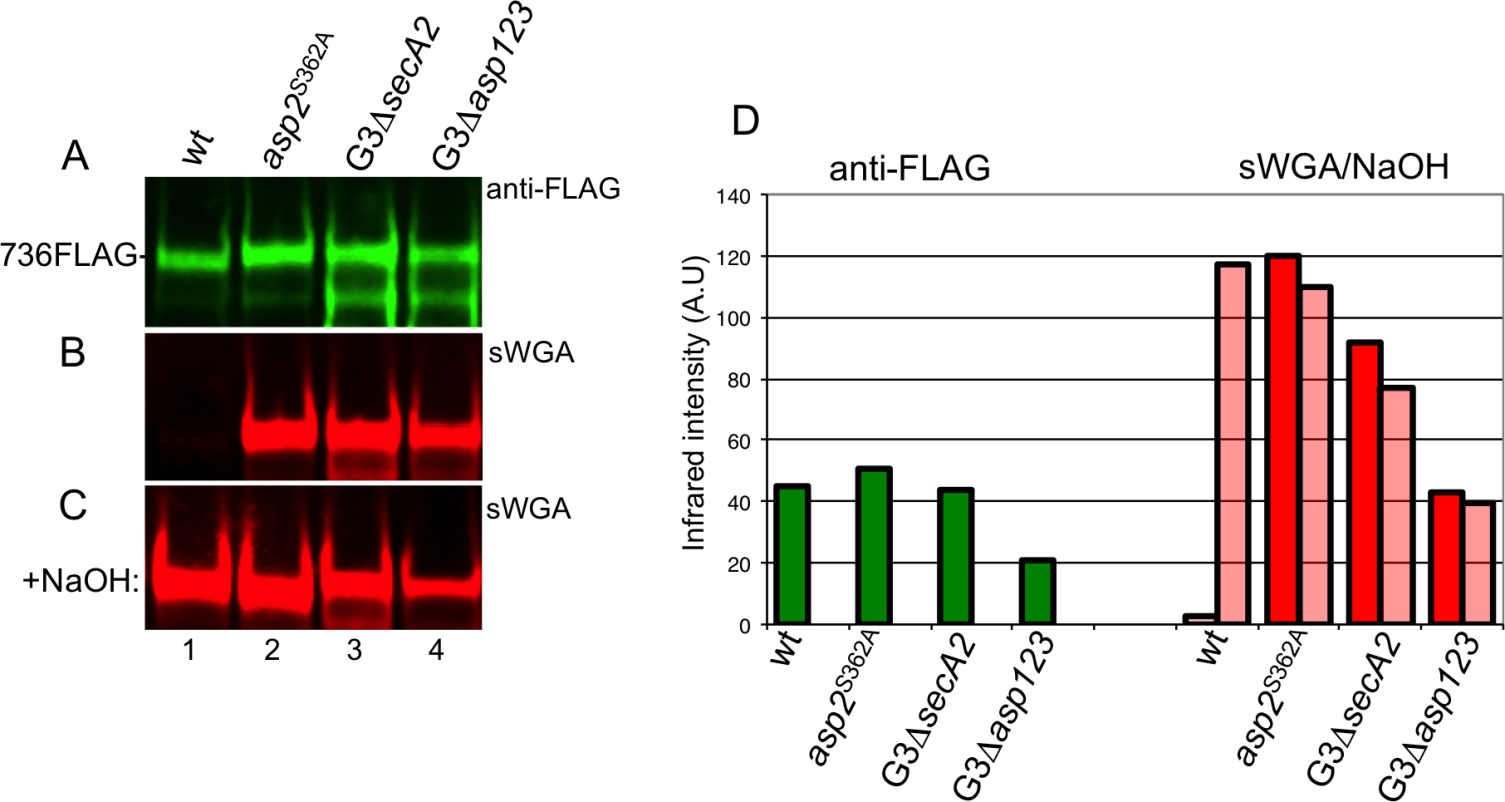
Assessment of anti-FLAG and sWGA reactivity of GspB736flag when exported through the general secretory pathway. A) Western blot analysis of GlcNAc-modified GspB736flag exported through the aSec system from the Δ*gly-nss* parental strain PS3309 (lane 1) and derivative strain harboring the Asp2 S362A mutation, PS3540 (lane 2), or when rerouted to the Sec pathway (via the *G3 signal sequence mutation [26]) by the Δ*gly-nss* accessory Sec deficient strains PS3316 (lane 3), PS3317 (lane 4). B) sWGA reactivity of GspB736flag exported via the aSec or Sec system in defined glycosylation backgrounds. C) GlcNAc reactivity of secreted GspB736flag following saponification. GlcNAc reactivity was assessed through lectin blot analysis using biotinylated sWGA as a GlcNAc probe. D) Densitometry analysis of GspB736flag levels (green bars) and GlcNAc reactivity before (red bars) and after saponification treatment (open red bars). The y-axis represents GspB736flag levels and GlcNAc reactivity based on band intensity analysis via LI-COR imaging. Western and lectin blot analysis of secreted GspB variants together with corresponding densitometry analysis are representative of at least three different genetic transformants of each strain analyzed.

We next asked whether Asp2-mediated acetylation of GspB was specific to the aSec pathway. To address this question, we compared the acetylation of GspB736flag transported via the aSec system, with that of GspB736flag*G3, which contains G75L/G79A/G80C mutations within the hydrophobic core of the signal peptide. This altered signal peptide re-routes the preprotein to the general Sec pathway [26]. The GspB variants were expressed in strains M99, M99*asp2*^*S362A*^, or in two strains deficient in aSec transport (M99Δ*secA2* and M99Δ*asp123*). Levels of GspB736flag*G3 transported via the general Sec pathway were comparable to those of GspB736flag transported by the aSec pathway, as measured by Western blotting with anti-FLAG. Of note, the sWGA reactivity of GspB736flag secreted through the general Sec pathway was similar to that of GspB736flag exported from an Asp2^S362A^ mutant (Fig 7B, lanes 3 and 4 versus 2), even when Asp2 was present (M99Δ*secA2*). Moreover, upon base treatment, GspB736flag secreted via the general Sec was resistant to saponification, displaying no significant changes in sWGA reactivity (Fig 7B, lanes 3 and 4 versus Fig 7C lanes 3 and 4). These findings show that GspB736flag exported via the general Sec pathway does not undergo acetylation, even when Asp2 is present. Instead, acetylation of GspB by Asp2 only occurs during aSec transport.

### O-acetylation of GspB is essential for bacterial binding to human platelets

We have previously shown that the Asp2-dependent modification of GspB is essential for M99 to bind to immobilized sialyl-T antigen [20]. This glycan is found on platelet glycoprotein GPIbα, and is the major ligand on platelets for GspB [27]. Reduced platelet binding via GspB is associated with decreased virulence in an animal models of infective endocarditis [1], [28]. To determine whether loss of acetylation of GspB affects this interaction, we directly compared the platelet binding by M99, an isogenic Asp2^S362A^ mutant, and other M99 variants expressing altered GspB glycoforms.

**Fig 8:**
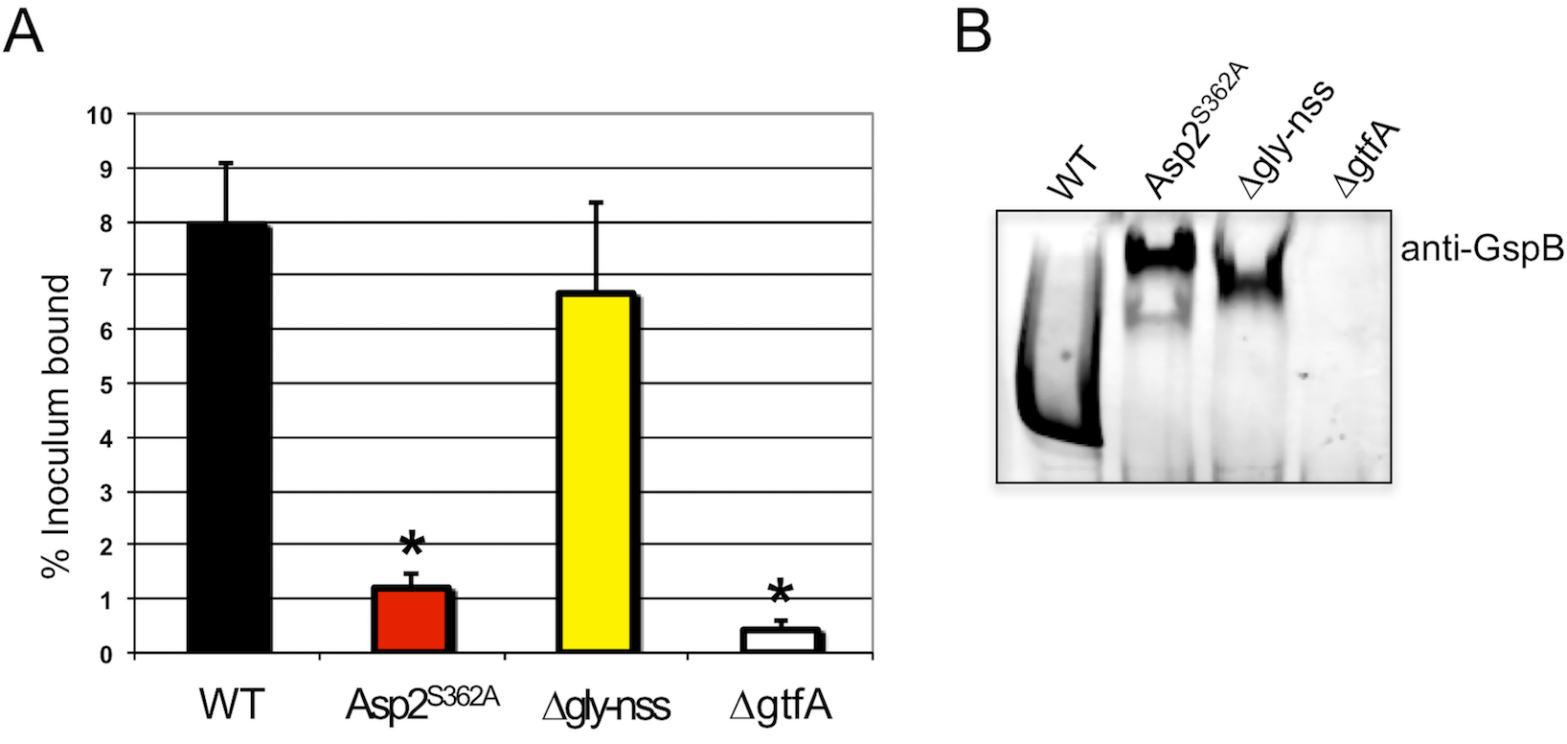
Binding of *S*. *gordonii* strains to human platelets. A) *S*. *gordonii* wild-type strain M99 and isogenenic mutants PS3536 (expressing the Asp2^S362A^ catalytic mutant), PS3319 (Δ*gly-nss*) and PS666 (Δ*gtfA*) expressing glycovariants of GspB were assessed for their binding to immobilized human platelets. Binding is expressed as the percent of input bacteria that remained bound to platelets after repeated washing of the wells. Bars indicate the means + S.D. of triplicate results from a representative experiment. * = *P* <0.01, compared with wild-type M99, one-way ANOVA, followed by post-hoc Tukey test. B) Expression of GspB and its glycoform variants on the surface of M99. Western blot analysis of cell fractions from the respective *S*. *gordonii* strains probed with anti-GspB polyclonal antisera, showing full-length GspB and its glycoform variants expressed on the surface of M99 at comparable levels. Of note, the Δ*gtfA* mutant does not express GspB on the bacterial surface due to instability of the protein from a loss of glycosylation [12].

As expected, M99 exhibited high levels of binding to human platelets, as compared with the Δ*gtfA* strain (PS666)(Fig 8A), which does not express GspB on the bacterial surface due to instability of the protein from a loss of glycosylation [12] (Fig 8B). Consistent with our previous reports, deletion of *gly* and *nss*, Δ*gly-nss* (PS3319) led to only a small decrease in platelet binding compared with M99. In contrast, M99 expressing the Asp2^S362A^ catalytic mutant (PS3536) had significantly reduced binding to platelets, comparable to those observed with the Δ*gtfA* deletion strain. These results indicate that the O-acetylation of GlcNAc moieties on GspB is essential for adhesive properties of the glycoprotein.

## Discussion

The biogenesis of the SRR adhesins is a complex process, involving both their intracellular glycosylation and export to the cells surface by the aSec system (reviewed in [16]). Studies in *S*. *gordonii* and *S*. *parasanguinis* have shown that glycosylation is mediated by a series of glycosyltransferases that sequentially add glycans to the SRR regions of the adhesin [13], [25]. The deposited glycan can range from a single *O*-linked GlcNAc residue to more complex glycan structures, depending on the number and type of Gtfs encoded within each SRR adhesin-aSec operon [13], [14]. Although the roles of the Asps in export are not fully defined, Asps1, 2 and 3 have been shown to enhance the engagement of the SRR preprotein with SecA2 [19], while Asp4 and Asp5 form a membrane complex with SecY2 [29].

Glycosylation and transport of the SRR adhesins have been viewed as independent processes, in part because SRR glycosylation can be reconstituted *in vitro* [14] [25] and because glycosylation of the SRR adhesin is not required for aSec transport [17]. However, our results demonstrate that the modification and transport of the SRR adhesins are linked by Asp2. In addition to its essential role in aSec transport, Asp2 functions as an enzyme mediating the O-acetylation of proximal GlcNAc residues within the SRR glycan. The catalytic activity of Asp2 was entirely dispensable for GspB export, thus demonstrating that Asp2 is a bi-functional protein mediating separate events in biogenesis.

Asp2 is found throughout all SRR adhesin-aSec operons but not in other loci, and its Ser-Asp-His catalytic triad is uniformly conserved [20]. These findings, along with the presence of O-acetylated GlcNAc moieties within the glycan of the Srr1 adhesin (one of two SRR adhesins expressed on the surface of *S*. *agalactiae*) [30], suggest that acetylation is likely to be a common glycan modification on SRR glycoproteins. It was previously hypothesized that the OatA/B system, which mediates the MurNAc/GlcNAc *O*-acetylation of bacterial peptidoglycan (PG) [31] [32], might be responsible for the acetylation of Srr1 [30]. Our findings indicate, however, that Asp2 is responsible for this modification. Indeed, mutagenesis of *S*. *gordonii* Asp2 resulted in the complete loss of O-acetylated GlcNAc on GspB, suggesting that Asp2 may be the sole *O*-acetyltransferase modifying the SRR adhesins.

The loss of GlcNAc O-acetylation had a profound effect upon adhesin function, where GspB-mediated streptococcal binding to human platelets was markedly reduced, to levels seen with a GspB-deficient strain. This differed significantly from what was seen with M99 lacking Nss or Gly modifications, where binding was minimally affected. The precise mechanism by which the loss of *O*-acetyl groups impairs the binding of M99 to platelets is as yet unknown. Since a non-glycosylated recombinant form of the binding-region of GspB binds to platelets with relatively high affinity [33], it is likely that acetylation of the SRR glycan is needed to maintain the proper conformation of GspB for binding to its platelet receptor. It is also possible that acetylation alleviates occlusion of the binding region caused by glycosylation of the neighboring SRR regions. We have previously shown that loss of GspB-mediated platelet binding (either by deletion of the adhesin or mutagenesis of the binding region) is associated with reduced virulence in animal models of streptococcal endocarditis [1] [28], and thus it is highly likely that acetylation of GspB is required for maximal pathogenicity. Indeed, as GspB *O*-acetylation is critical for platelet binding, Asp2 could provide an effective antimicrobial target, as has been proposed for other bacterial *O-*acetyltransferases linked to virulence [31], [23].

Our finding that the O-acetylation of GspB was present only when the substrate had undergone aSec transport indicates that these two processes are coordinated, with Asp2 serving as a nexus for biogenesis. As glycan O-acetylation is necessary for optimal GspB function, the coordination of this modification with transport may serve to assure that the SRR adhesion is fully functional. Moreover, analysis of glycopeptides from secreted GspB736flag revealed that not all GlcNAc residues were O-acetylated (Table 1), further suggesting that there may be a requirement to limit or regulate this modification.

These results also provide additional insights as to why a dedicated transporter is needed for the SRR adhesins. The general Sec system is responsible for exporting a large number and variety of substrates, the export of some being essential for cell viability [34]. It is possible that the requirement to coordinate a post-translation modification with export via the Sec pathway would impede export of other Sec substrates, which could potentially be detrimental to the cell. Thus, having a dedicated transporter enables the cell to alleviate any undue pressure upon its secretome. It is also possible that O-acetylated glycan is incompatible with engagement of the Sec machinery. Our findings show that the Sec system can export a non-acetylated SRR glycoform (Fig 7). However, just as O-acetylation affects GspB binding to its host ligand, this modification could prevent interactions with the Sec machinery. The latter would explain in part the evolution of a SecA2/SecY2 paralogue, to accommodate an acetylated substrate.

**Fig 9:**
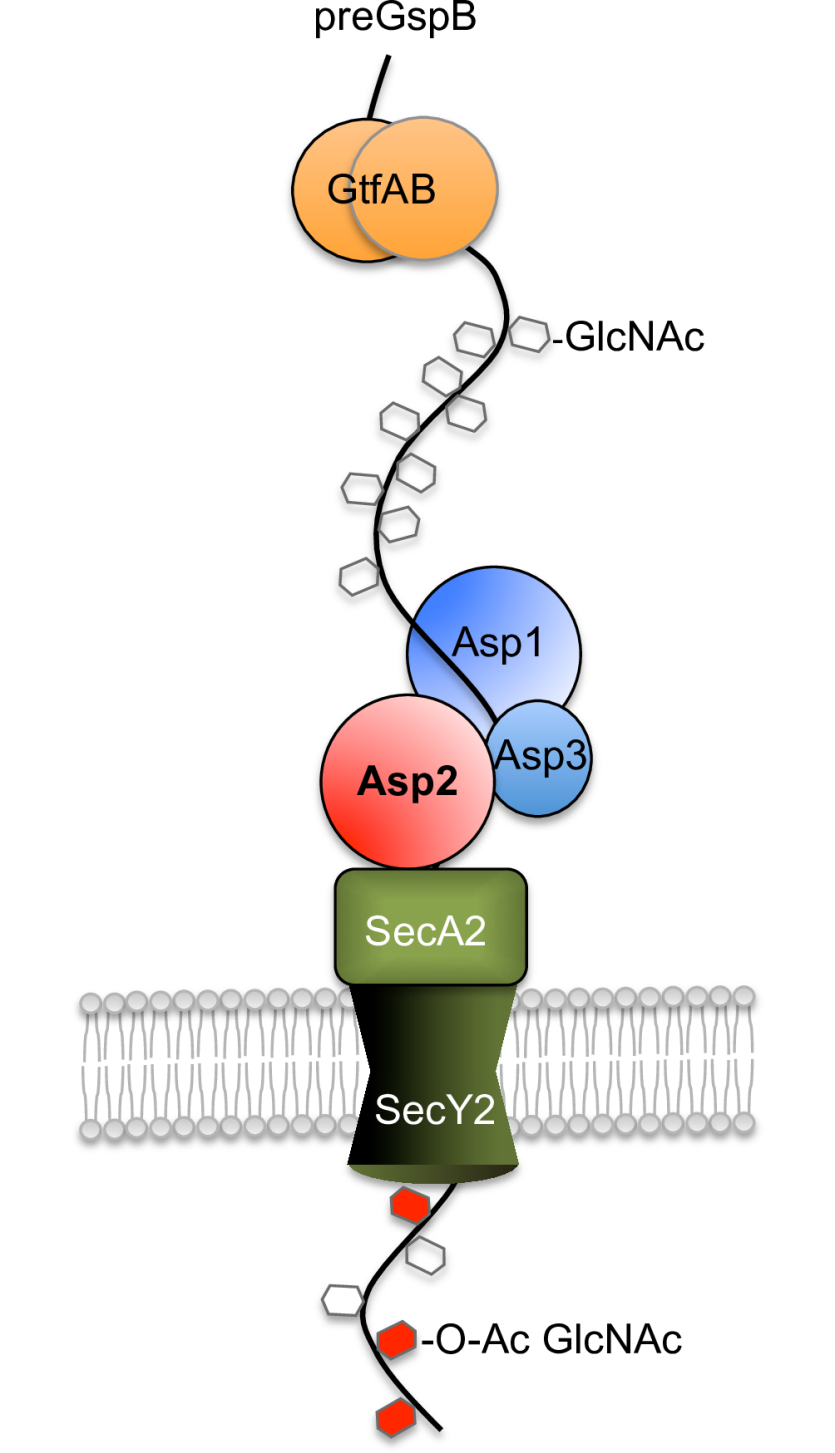
Proposed model for transport-mediated O-acetylation of GspB. GspB is glycosylated by the GtfAB complex to deposit GlcNAc along the SRR regions [25], [13]. Glycosylated GspB maybe delivered to SecA2 via an interaction with one or more Asps [18], [19]. Asp3 binds both Asp1 and Asp2 forming a protein complex [17] localizing at the membrane [39]. Collectively, the Asps enhance the interaction between the GspB preprotein and SecA2 enabling full substrate engagement with the translocation (SecA2/SecY2) machinery [16], [19]. Following full engagement of aSec translocation, GspB is O-acetylated by Asp2 (GspB glycosylation by Nss and Gly have been omitted for clarity).

We have previously shown Asp1-3 can localize with SecA2 at the bacterial membrane to facilitate translocation (reviewed in [16]) and our current findings suggest that Asp2 acetylates GspB at this same location (Fig 9). These findings, along with the absence of O-acetylated glycoforms within the cytosol, suggest that acetylation occurs at the membrane and in association with aSec transport. Collectively, our results indicate the aSec system is a highly specialized export and modification system, where linking of these two processes via Asp2 ensures that the correct SRR glycofrom is expressed on the bacterial surface. The coupling of glycan O-acetylation with transport may serve as means by which this critical modification can be more precisely controlled, and may further explain why the aSec system has evolved as a separate protein secretion system. The roles of other aSec components towards SRR glycoprotein O-acetylation and export are currently under active investigation.

## Methods and materials

### Ethics statement

Human platelets were collected from volunteers, under a protocol approved by the UCSF Committee on Human Research (IRB number 11–06207).

### Bacterial strains and plasmids

The bacterial strains and plasmids used in this study are listed in Table 2. *S*. *gordonii* strains were grown in Todd-Hewitt broth (THB, Becton, Dickinson and Company) or on 5% sheep blood agar (Hardy Diagnostics) at 37°C in a 5% CO_2_ environment. Antibiotics were added to the media at the following concentrations: 60 μg mL^-1^ erythromycin and 100 μg mL^-1^ spectinomycin. *E*. *coli* strains XL1-Blue and BL21(DE3) were grown in Luria-Bertani (LB) broth or on LB agar containing 30 μg mL^-1^ kanamycin, 100 μg mL^-1^ ampicillin, 50 μg mL^-1^ spectinomycin or 300 μg mL^-1^ erythromycin when appropriate.

**Table 2:** Bacterial strains and plasmids.

Strain or plasmid	Genotype or description	Reference
*S*. *gordonii*		
M99	endocarditis causing parental strain	[37]
PS1225	M99 expressing *gspB*736flag	[20]
PS1244	PS1225 Δ*asp2*; Spec^R^	[20]
PS3539	PS1225 *asp2*^S362A^	This study
PS3309	PS1225 Δ*gly-nss*; Spec^R^	This study
PS3540	PS3539 Δ*gly-nss*, *asp2*^S362A^; Spec^R^	This study
PS921	M99 expressing *gspB*1060flag	[10]
PS3349	PS921 Δ*asp2*; Spec^R^	This study
PS3541	PS921 *asp2*^S362A^	This study
PS3318	PS921 Δ*gly-nss*; Spec^R^	This study
PS3542	PS3541 Δ*gly-nss asp2*^S362A^; Spec^R^	This study
PS1290	PS1225 Δ*gtfA*; Spec^R^	This study
PS3549	PS1225 Δ*gtfA*, *asp2*^S362A^; Spec^R^	This study
PS1064	PS921 Δ*gtfA*; Spec^R^	This study
PS3550	PS921 Δ*gtfA*, *asp2*^S362A^; Spec^R^	This study
PS3551	PS1225 *asp2*^E452A^	This study
PS3552	PS1225 *asp2*^H482A^	This study
PS3553	PS1225 *asp2*^S362AE452A^	This study
PS3554	PS1225 *asp2*^S362AE452AH482A^	This study
PS3555	PS921 *asp2*^E452A^	This study
PS3556	PS921 *asp2*^H482A^	This study
PS3557	PS921 *asp2* ^S362AE452A^	This study
PS3558	PS921 *asp2*^S362AE452AH482A^	This study
PS3584	PS1225 Δ*asp2*,Δ*gly-nss*; Cm^R^, Spec^R^	This study
PS1226	PS1225 Δ*secA2*	[38]
PS3311	PS1225 Δ*gly-*Δ*asp3*; Spec^R^	This study
PS3310	PS1226 Δ*gly-nss*; Spec^R^	This study
PS3605	PS1226 Δ*gly-nss asp2*^S362A^; Spec^R^	This study
PS922	PS921 Δ*secA2*	[10]
PS3323	PS922 Δ*gly-*Δ*asp3*; Spec^R^	This study
PS3322	PS922 Δ*gly-nss*; Spec^R^	This study
PS3606	PS922 Δ*gly-nss asp2*^S362A^; Spec^R^	This study
PS3316	PS1225 *gspB*736flag L75A79C80 Δ*gly-nss*, Δ*secA2*; Spec^R^	This study
PS3317	PS1225 *gspB*736flag L75A79C80 Δ*gly*-Δ*asp3*; Spec^R^	This study
PS666	M99 Δ*gtfA*; Spec^R^	[12]
PS3319	M99 Δ*gly-nss*; Spec^R^	This study
PS3536	M99 *asp2*^S362A^	This study
*E*. *coli*		
TOP10	Host cell for cloning	Invitrogen
BL21	Host cell for protein expression	Novagen
PS875	*E*. *coli* expressing glycosylated GST-SRR1	[25]
Plasmids		
pCOLA.H6*asp123*	*E*. *coli* expression vector encoding *asp123* Kn^R^	This study
pMalE-*asp2*-H6	*E*. *coli* expression vector encoding MalE-*asp2* fusion protein; Amp^R^	This study
pMalE*-asp2*^*S362A*^-H6	*E*. *coli* expression vector encoding MalE-*asp2*^S362A^ fusion protein; Amp^R^	This study
pGLYNSSK	pS326 carrying upstream fragments of *gly* and downstream fragments of *nss*; Spec^R^	This study
pGLYNSSK	pS326 carrying upstream fragments of *gly* and downstream fragments of *asp3*; Spec^R^	This study

Cm^R^, chloramphenicol resistant

Erm^R^, erythromycin resistant

Spec^R^, spectinomycin resistant

### DNA manipulations

Routine molecular biology techniques for cloning, sequencing and PCR amplification were performed by as described previously [35]. Chromosomal DNA was isolated from *S*. *gordonii* according to Madoff *et al*. (1996) [36]. Plasmid DNA was isolated from *E*. *coli* using miniprep columns (Qiagen). DNA restriction and modification enzymes were used according to manufacturer’s recommendations (NEB). *E*. *coli* cells were transformed following CaCl_2_ treatment, while *S*. *gordonii* was transformed as described previously [9].

### Site-directed mutagenesis

Mutagenesis of *asp2* was conducted using the QuikChange Lightning site-directed mutagenesis kit (Agilent Technologies) as previously described [20] and either pET.H6Asp2 or pCOLA.H6*asp*123 as the template. Following PCR, *Dpn* I was added to the reaction mixture to remove the original methylated plasmid. The remaining plasmids in the reaction mixture were then used to transform *E*. *coli* XL1-Blue, and the resulting clones were screened for the correct point mutations by DNA sequencing (Sequetech DNA, Mountain View).

### Construction of Asp2 catalytic mutant strains

Point mutations within the *asp2* gene of *S*. *gordonii* strain M99 were achieved through allelic replacement. Using primers previously described [20], the codons Ser-362, Glu-452 and His-482 of *asp2* were replaced with those for Ala by site-directed mutagenesis using the plasmid pCOLA.H6*asp123* (encoding *asp123*) as a template. Plasmids were then used to transform M99 Δ*asp2*::*spec* strains expressing either GspB736flag or GspB1060flag, resulting in a replacement of the spectinomycin cassette with the mutated *asp2*. Transformants were plated on sheep blood agar plates and scored for the loss of spectinomycin resistance. Chromosomal DNA was isolated from spectinomycin sensitive clones and the *asp2* gene was PCR amplified and sequenced to confirm the correct mutant replacement.

### Construction of GspB736flag*G3 mutant strains

The construction of the GspB736flag*G3 (i.e. G75L/G79A/G80C) signal peptide mutant strain PS1129 and the GspB736flag*G3 Δ*secA2* strain PS1146 have previously been described [26]. Replacement of the *gly-nss* genes within PS1146 by a *spec* cassette was performed using a modification of the method used to delete *gly* or *nss* individually [12]. In brief, a chromosomal segment upstream of *gly* was amplified by PCR using primers glyKO4 and glyKO5, and a chromosomal segment downstream of *nss* was amplified using primers nssKO3 and nssKO5. The fragments were cloned upstream or downstream, respectively, of the *spec* gene in pS326. The resulting plasmid, pGLYNSSK, was introduced to PS1146 by natural transformation, and allelic replacement was monitored by selection on spectinomycin. Similarly, replacement of the *gly-asp3* genes in PS1129 with a *spec* cassette was accomplished by replacing the upstream chromosomal segment of *asp3* in pORF3K [12] with that of the chromosomal segment upstream of *gly* as described above. The resulting plasmid, pGLYASP3K, was introduced to PS1146 by natural transformation, and allelic replacement was monitored by selection on spectinomycin.

### Analysis of secreted and protoplast proteins

Overnight cultures of M99 were diluted 1:6 in fresh THB, grown for 5 hr at 37°C, and the cells harvested by centrifugation. For analysis of secreted proteins, samples of clarified culture media were either mixed with protein sample buffer (Novagen) prior to SDS-PAGE separation and Western blot analysis or used directly in saponification analysis. For analysis of non-exported proteins, pelleted cells were resuspended in protoplast lysis buffer (PLB: 60 mM Tris pH 6.8, 150 mM NaCl, 10% raffinose and 0.5U μL^-1^ mutanolysin). The PLB suspensions were incubated for 1 hr at 37°C, boiled in protein sample buffer, followed by SDS-PAGE and Western blot analysis. For saponification analysis of non-exported proteins, pelleted cells were suspended in lysis buffer (LB: 60 mM Tris pH 7, 150 mM NaCl) and lysed using a MiniBeadbeater (Biospec) by using 2× 60-s bursts at RT at full speed. Insoluble material was pelleted by centrifugation, and lysates were normalized for protein concentration prior to saponification (see below).

### GspB736flag purification

Secreted GspB736flag was purified from M99 culture supernatants (14 Liters) as previously described [10]. In brief, M99 strains PS3309 and PS3540 were grown overnight in THB and cells removed by centrifugation. Proteins secreted into the culture media were precipitated overnight in ammonium sulfate (NH4)_2_SO_4_ (25% final concentration), recovered by centrifugation and reconstituted in Tris buffered saline (TBS: 50 mM Tris pH 7.5, 150 mM NaCl). Glycosylated GspB736flag was subsequently purified from TBS under native conditions by affinity chromatography using sWGA agarose (Vector) and eluted in 300 mM GlcNAc. GspB736flag fractions were pooled, concentrated by ultrafiltration using an Amicon Ultra centrifugal filter device (100 kDa cutoff) and reconstituted in dH_2_O until further analysis.

### Densitometry analysis

To quantify differences in GspB transport and glycosylation, blots of GspB736flag or GspB1060flag were incubated at room temperature (RT) simultaneously with mouse anti-FLAG antibody (Sigma) and sWGA (Vector) used at concentrations of 1:5000 and 0.4 μg mL^-1^ respectively. Blots were incubated for 2 hr, followed by another 90 min incubation with a 1:20,000 dilution of both HiLyte Fluor800-anti-Mouse IgG and HiLyte Fluor680-streptavidin (Anaspec). Immunoreactive bands were visualized using an infrared imager (LI COR Biosciences) at both 680 nm and 800 nm. Band intensity was analyzed using Odyssey v3.0 software.

### GspB saponification

Base-promoted ester-hydrolysis was achieved through mild NaOH treatment. Culture supernatants containing secreted GspB variants were buffered in 10 mM Tris pH 7 and incubated with 100 mM NaOH for 1 hr at 37°C. Protoplast generated by beadbeating were clarified by centrifugation at 14,000 rpm for 10 min and the supernatants were incubated with 100 mM NaOH for 1 hr at 37°C.

### Quadrupole time-of-flight (Q-TOF) Liquid chromatography/Mass spectrometry (LC/MS) analysis of GspB736flag glycopeptides

GspB736flag glycoforms from both Δ*gly*-*nss* and Δ*gly*-*nss* Asp2^S362A^ backgrounds were overproduced and then purified by affinity chromatography on sWGA-agarose as described above. To produce glycopeptides suitable for LC/MS analysis, both GspB glycoforms (10 μg) were prepared by in-gel digestion using 10% (*v/v*) acrylamide gels. Following SDS-PAGE and staining with Coomassie Brilliant Blue, bands corresponding to glycosylated GspB were extracted using a clean scalpel and destained with 50% (*v/v*) acetonitrile (ACN)/25mM ammonium bicarbonate, pH 7.5 and placed into microfuge tubes. The destained gel slices were then dehydrated with ACN and dried *in vacuo* using a centrifugal evaporator. The slices were resuspended in 50 mM ammonium bicarbonate, pH 7.5 and treated with both sequencing grade trypsin/Lys-C protease mix (20 μg; Promega, Madison, WI) and endoproteinase Glu-C (20 μg; Thermo-Pierce, Waltham, MA). Following digestion for 18 h at 37°C, peptides were recovered from the slices by sonication for 10 min in a water bath sonicator followed by vortexing for 5 min. The acrylamide gel was pelleted by centrifugation (1000 x *g*, 1 min), and the recovered supernatants were dried in a centrifugal evaporator.

The recovered glycopeptide/peptide mixtures were dissolved in 15 μL water and analyzed by LC/MS using an Agilent 1200 LC coupled to an Agilent UHD 6530 Q-TOF mass spectrometer (Agilent, Santa Clara, CA). Samples were loaded onto the C18 column (Agilent AdvanceBio Peptide Map; 100 mm × 2.1 mm; 2.7 μm) previously equilibrated with solvent A (water, 2% ACN, 0.1% formic acid) and they were resolved using a linear step-gradient of 0–45% B (ACN, 0.1% formic acid) over 40 min, then 45–55% B over 10 min, followed by a wash step at 95% B at a flow rate of 0.2 mLmin^-1^. The Q-TOF was operated in extended dynamic range positive-ion auto MS/MS modes with an m/z range of 300–2000 *m/z* and a capillary voltage of 4 kV. Three precursor ions were chosen for collision induced dissociation (CID) fragmentation. To identify GspB, the MS data were analyzed using PEAKS 7 software (Bioinformatics Solutions Inc., Waterloo, ON). Glycopeptides and their *O*-acetylated forms were identified with MassHunter Qualitative Analysis software (Agilent, Santa Clara, CA) by manually searching the MS/MS data for the presence of diagnostic HexNAc and *O*-acetylHexNAc oxonium fragmentation product ions (*m/z* = 204.08 and 246.09, respectively).

### GST-SRR1 glycan profiling

We have previously shown that a GST-GspB fusion encompassing the entire SRR1 region of GspB (GST-SRR1) can be glycosylated by all the glycosyltransferases within the GspB operon when reconstituted in *E*. *coli* [20]. Moreover, this GST-GspB fusion proved to be highly soluble and easily purified from *E*. *coli*, and was therefore considered representative of the GspB glycan and suitable for subsequent glycan profile analysis. Glycosylated GST-SRR1 proteins were GST purified as described below and subjected to glycan profiling through MALDI-TOF Mass spectrometry profiling, performed as a service by the Glycotechnology Core Resource at the University of California, San Diego. In brief, glycans were released from GST-SRR1 by reductive beta elimination using base-borohydride treatment. The O-glycans were then purified and per-methylated and dissolved in MeOH. Dissolved permethylated glycans were mixed with super-DHB matrix in a 1:1 (v/v) ratio and spotted on a MALDI plate and MALDI-TOF MS analysis was performed in positive ion mode. The proposed structures for mass peaks were extracted from the CFG database using GlycoWorkbench software.

### Cloning, overexpression and purification of MalE-Asp2H6 and its derivatives

For inducible expression in E. coli, *asp2* and its catalytic mutant (*asp2*^S362A^) were cloned into the expression vector pMAL-c2x (NEB) resulting in an in-frame fusion of MalE at the N-terminal of Asp2 and a His_6_ -tag (H6) at the C-terminus. The pMal-*asp2*-H6 constructs were introduced to *E*. *coli* BL21 (Lucigene) and grown in Low Salt LB medium containing 2% glucose at 37°C supplemented with ampicillin. Upon reaching an OD_600_ of 0.6, the cells were induced with 1 mM IPTG and allowed to grow for an additional 18 h at 17°C. The cells were resuspended in Lysis buffer (20 mM Tris pH 8.5, 200 mM NaCl, 1 mM EDTA and 0.5% Triton-X100) supplemented with lysozyme (50 μg mL^-1^) and lysed by passage through a French press cell (15, 000 psi). MalE-Asp2-H6 fusion proteins were purified from clarified lysates under native conditions by affinity chromatography using amylose resin (NEB). Further purification of MalE-Asp2-H6 was achieved by affinity purification using Ni^2+^-nitrilotriacetic acid agarose (Qiagen). Semi-purified MalE-Asp2-H6 protein was reconstituted in His6-binding buffer (50mM Sodium phosphate, pH 8, 150 mM NaCl and 10 mM imidazole) and mixed with pre-equilibrated resin under constant rotation at 4°C. Mal-Asp2_H6_ proteins were eluted in His6-binding buffer containing 300 mM imidazole, concentrated by ultrafiltration using an Amicon Ultra centrifugal filter (100 kDa cutoff) and dialyzed against 50 mM Sodium phosphate, pH 7 containing 150 mM NaCl overnight at 4°C before use.

### Expression and purification of glycosylated SRR protein

Construction of a *gstSRR1*-*gtfAB* co-expression plasmid, resulting in the expression a GlcNAc-glycosylated GST-SRR1 protein has been described previously [26]. *E*. *coli* strains transformed with this construct (PS875) were grown to an OD_600_ of 0.6 in LB medium at 37°C supplemented with ampicillin and induced as described above. Cells were resuspended in GST-Lysis buffer (50 mM Tris pH 8, 150 mM NaCl and 0.5% Triton X-100) supplemented with lysozyme (50 μg mL^-1^) and lysed by sonication. GST-SRR1 fusion protein was purified from clarified lysates under native conditions by affinity chromatography using glutathione agarose (Pierce) according to the manufacturer’s instructions. Purified GST-SRR1 protein was eluted in 50 mM Sodium phosphate, pH 7 containing 150 mM NaCl and 30 mM glutathione and concentrated and dialyzed as described above.

### Enzymatic assay of Asp2

The *in vitro* acetylesterase activity of Asp2 was assessed as described previously [24]. In brief, reaction mixtures contained 2 mM *p*-nitrophenyl acetate (*p*NP-Ac) (dissolved in ethanol, 2% final) (Sigma), 10 μg of MalE-Asp2-H6 or its mutant form and 10 μg of GtfAB glycosylated GST-SRR1 in a total volume of 200 μL in 50 mM Sodium phosphate, pH 7. Reactions, performed in triplicate, were initiated by the addition of *p*NP-Ac, and were monitored continuously at 405 nm for the release of *p*NP over 30 min at 25°C using a Spectra Max 250 microplate reader (Molecular Devices). The hydrolysis of *p*NP-Ac by Asp2 results in the release of *p*NP which was monitored as an increase of absorbance at 405 nm. Spontaneous *p*NP-Ac hydrolysis was subtracted from all enzyme catalyzed reactions at time zero. A calibration curve for *p*NP was obtained under the reaction conditions and used to calculate rate of *p*NP release.

### Binding of *S*. *gordonii* to platelet monolayers

The binding of *S*. *gordonii* to immobilized platelets was performed as described previously [9]. In brief, strains were grown for 18 hr, washed twice in Dulbeccos PBS (DPBS), sonicated briefly to disrupt aggregated cells and diluted to approximately 2x10^7^ cfu mL^-1^. Bacterial suspensions were applied to wells of a microtiter plate coated with human platelets. After a 2 hr incubation at room temperature, the unbound bacteria were removed by aspiration. Wells were washed three times with DPBS and the bound bacteria were released by trypsinization. The number of input and bound bacteria were determined by plating serial dilutions of bacterial suspensions on sheep blood agar plates, and the binding was expressed as the percent of the input bound to human platelets. The differences in binding between groups were examined by one-way ANOVA with post-hoc Tukey HSD (honestly significant difference) (http://vassarstats.net/anova1u.html). *P* < 0.01 was considered statistically significant.

## Supporting information

S1 Fig MALDI-TOF mass spectrum profile of per-*O*-methylated O-glycan from gstSRR1 glycosylated by GtfAB and Nss. A single glycan at m/z 534.19 was identified from the spectrum of released permethylated glycans representative of a GlcNAc-Glc disaccharide. Proposed glycan structures are shown.
(TIFF)

S2 Fig MALDI-TOF/TOF mass spectrum of per-*O*-methylated O-glycan from gstSRR1 glycosylated by GtfAB, Nss and Gly. A single glycan at m/z 738.34 was identified from the spectrum of released permethylated glycans representative of a GlcNAc-Glc-Glc trisaccharide. Proposed glycan structures are shown.
(TIFF)

S3 Fig Asp2 does not deposit GlcNAc directly on GspB. Western blot analysis of GspB736flag and GspB1060flag export by parental strains PS1225 (lane 1) and PS921 (lane 4), their Δ*gtfA* strains PS1290 (lane 2) and PS1064 (lane 5) and their corresponding Δ*gtfA* derivative strains harboring the S362A mutation within *asp2*, PS3549 (lane 3), PS3550 (lane 6). Culture media was collected from exponentially growing strains and proteins were separated by SDS-PAGE and subjected to Western blot analysis using anti-FLAG antibodies and biotinylated sWGA to determine GspB levels and GlcNAc reactivity, respectively.
(TIFF)

S4 Fig MS/MS spectra of GspB736flag glycopeptide (^660^SASTSSSVSASE^671^) generated by CID. (A) Fragmentation spectrum of peak 2, m/z = 773.31 [M+3H]^3+^ O-glycosylated at six positions. (B) Fragmentation spectrum peak 2*, m/z = 787.32 [M+3H]^3+^ O-glycosylated at six positions and O-acetylated at one position. *O*-GlcNAc modifications are designated by a blue square. MS spectra of precursor ions are shown in the insets.
(TIFF)

S5 Fig MS/MS spectra of GspB736flag glycopeptide (^672^SASTSASVSASE^683^) generated by CID. (A) Fragmentation spectrum of peak 3, m/z = 767.98 [M+3H]^3+^ O-glycosylated at six positions. (B) Fragmentation spectrum of peak 3*, m/z = 781.98 [M+3H]^3+^ O-glycosylated at six positions and O-acetylated at one position. *O*-GlcNAc modifications are designated by a blue square. MS spectra of precursor ions are shown in the insets.
(TIFF)

S6 Fig MS/MS spectra of GspB736flag glycopeptide (^624^SASASLVTSK^633^) generated by CID. (A) Fragmentation spectrum of peak 4, m/z = 588.27 [M+3H]^3+^ O-glycosylated at four positions. (B) Fragmentation spectrum of peak 4*, m/z = 902.91 [M+2H]^2+^ O-glycosylated at four positions and O-acetylated at one position. *O*-acetyl modifications are designated by a blue square. MS spectra of precursor ions are shown in the insets.
(TIFF)

S7 Fig Extracted ion chromatograms of m/z 204.08 ± 0.01 (GlcNAc; black) and m/z 246.09 ± 0.01 (*O*-acetylGlcNAc; red) of MS/MS spectra. (TIFF)

S8 Fig Extracted ion chromatograms of m/z 204.08 ± 0.01 of MS/MS spectra from GspB736FLAG (black) and base-treated GspB736FLAG (red) generated from a Δ*gly*Δ*nss* background. (TIFF)

S9 Fig *In vitro* O-acetylation of GspB. Western blot analysis of glycosylated SRR1 (lane 1), or glycosylated SRR1 co-incubated with *p*NP-Ac and MalE-Asp2-H6 (lane 2) (as described in methods and materials). O-acetylation *in vitro* reactions were stopped after 1 hr incubation by mixing with protein sample buffer (Novagen). Proteins were separated by SDS-PAGE and subjected to Western blot analysis using anti-GST antibodies or lectin blot analysis using biotinylated sWGA to determine glycosylated SRR1 levels and GlcNAc reactivity, respectively.
(TIFF)

S10 Fig Loss of an Asp2 modification does not impair GspB1060flag transport. Western blot analysis of Asp2-dependent export of GspB1060flag by *S*. *gordonii* Δ*asp2* strain PS3349 (lane 1), parental strain PS921 (lane 2) and derivative strains harboring the designated alanine substitution within the catalytic triad of Asp2, PS3541 (lane 3), PS3555 (lanes 4), PS3556 (lane 5), PS3557 (lane 6), PS3558 (lane 7). Culture media was collected from exponentially growing strains. Proteins were separated by SDS-PAGE (3–8%) and analyzed by Western blotting, using anti-FLAG antibody to detect GspB736flag.
(TIF)

S1 Table List of ions detected by Q-TOF LC/MS & location of glycopeptides within GspB736FLAG. (A) List of ions identified by Q-TOFF LC/MS from each of the four major glycopeptide fragments generated from GspB736FLAG protease digestion (Peaks 1–4). (B) Glycopeptide fragment location within the GspB736FLAG amino acid sequence. The first and second glycosylated serine rich repeat regions (SRR1 and SRR2) within GspB are shown in green and red respectively. Glycopeptide fragments corresponding to peaks 1–4 are highlighted in blue.
(TIF)
