# Supplementary figures and images for: O-acetylation of the serine-rich repeat glycoprotein GspB is coordinated with accessory Sec transport
Source: PLoS Pathog. 2017 Aug 21;13(8):e1006558. doi: 10.1371/journal.ppat.1006558 (PMC5578698; doi:10.1371/journal.ppat.1006558)

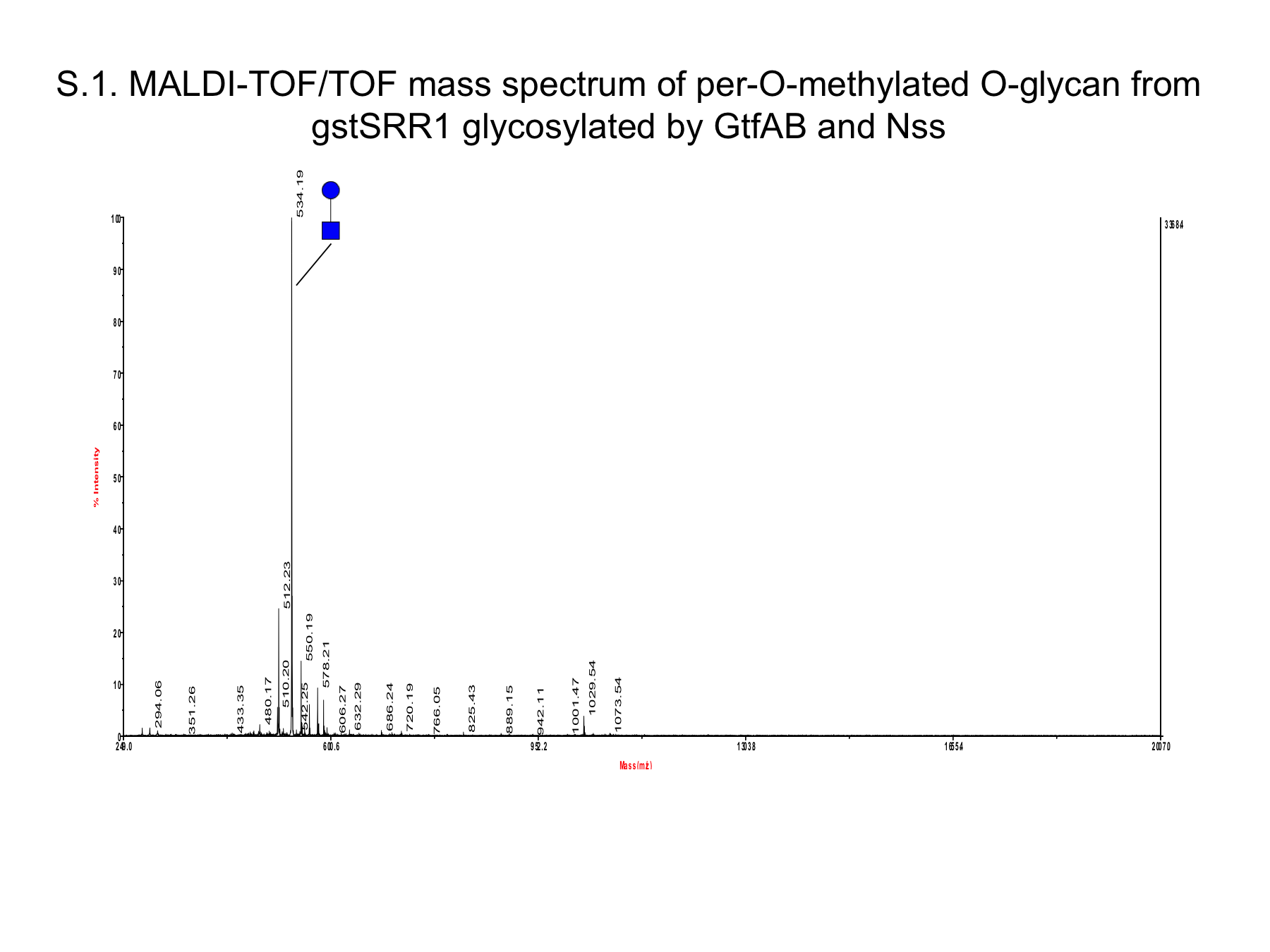

Supplement: S1 Fig — A single glycan at m/z 534.19 was identified from the spectrum of released permethylated glycans representative of a GlcNAc-Glc disaccharide. Proposed glycan structures are shown. (TIFF) [file ppat.1006558.s001.tiff]

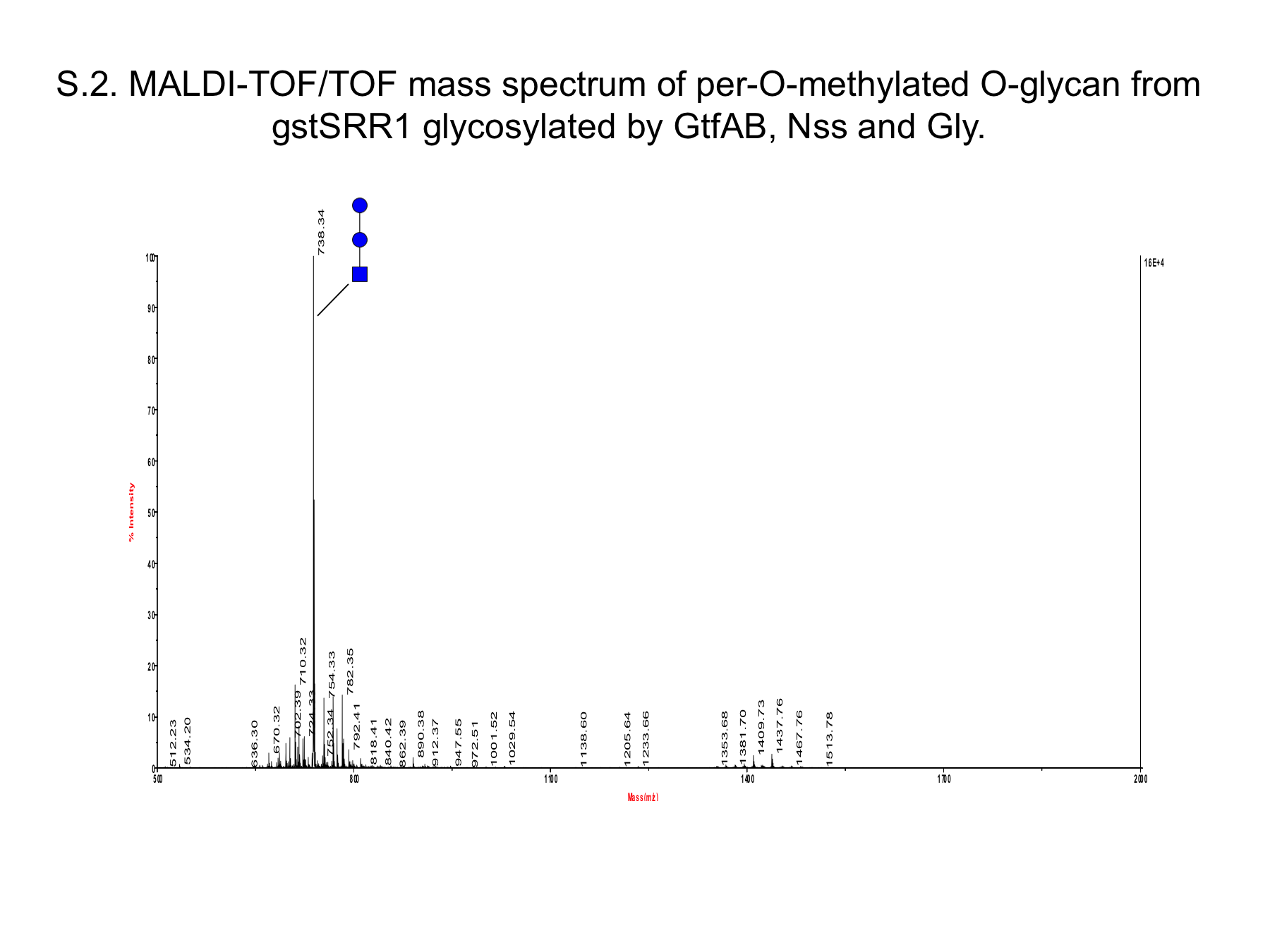

Supplement: S2 Fig — A single glycan at m/z 738.34 was identified from the spectrum of released permethylated glycans representative of a GlcNAc-Glc-Glc trisaccharide. Proposed glycan structures are shown. (TIFF) [file ppat.1006558.s002.tiff]

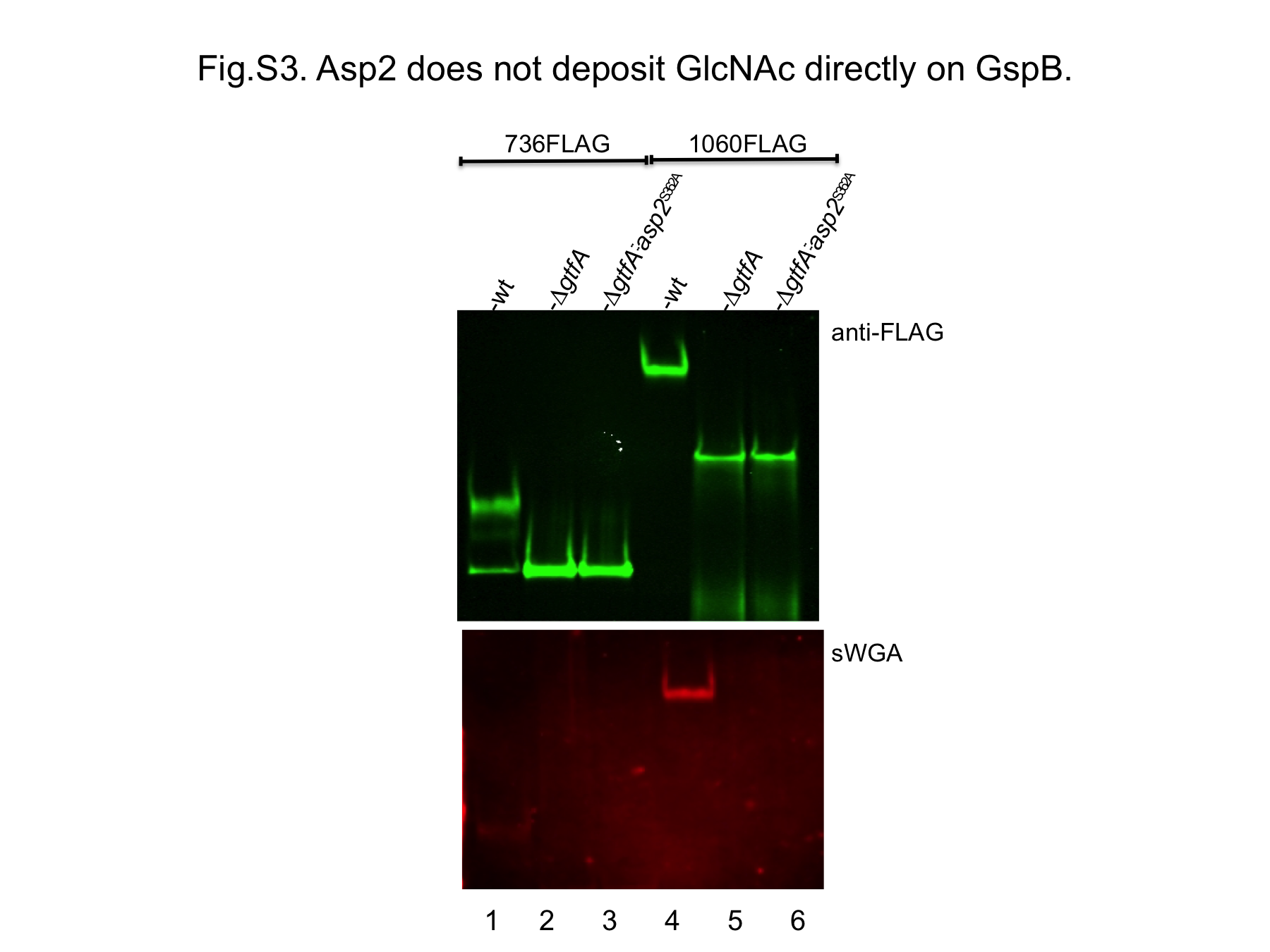

Supplement: S3 Fig — Western blot analysis of GspB736flag and GspB1060flag export by parental strains PS1225 (lane 1) and PS921 (lane 4), their ΔgtfA strains PS1290 (lane 2) and PS1064 (lane 5) and their corresponding ΔgtfA derivative strains harboring the S362A mutation within asp2, PS3549 (lane 3), PS3550 (lane 6). Culture media was collected from exponentially growing strains and proteins were separated by SDS-PAGE and subjected to Western blot analysis using anti-FLAG antibodies and biotinylated sWGA to determine GspB levels and GlcNAc reactivity, respectively. (TIFF) [file ppat.1006558.s003.tiff]

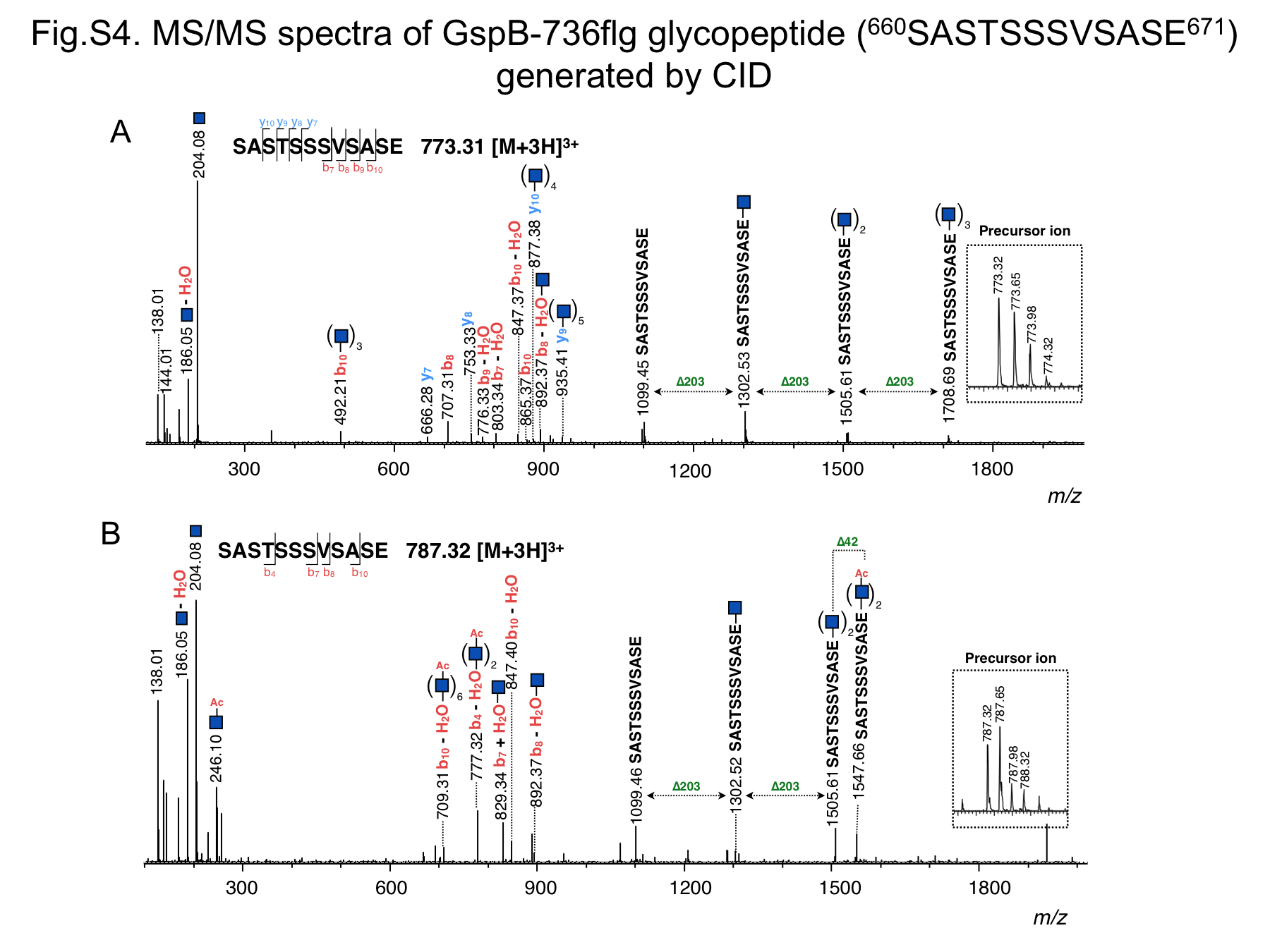

Supplement: S4 Fig — (A) Fragmentation spectrum of peak 2, m/z = 773.31 [M+3H]3+ O-glycosylated at six positions. (B) Fragmentation spectrum peak 2*, m/z = 787.32 [M+3H]3+ O-glycosylated at six positions and O-acetylated at one position. O-GlcNAc modifications are designated by a blue square. MS spectra of precursor ions are shown in the insets. (TIFF) [file ppat.1006558.s004.tiff]

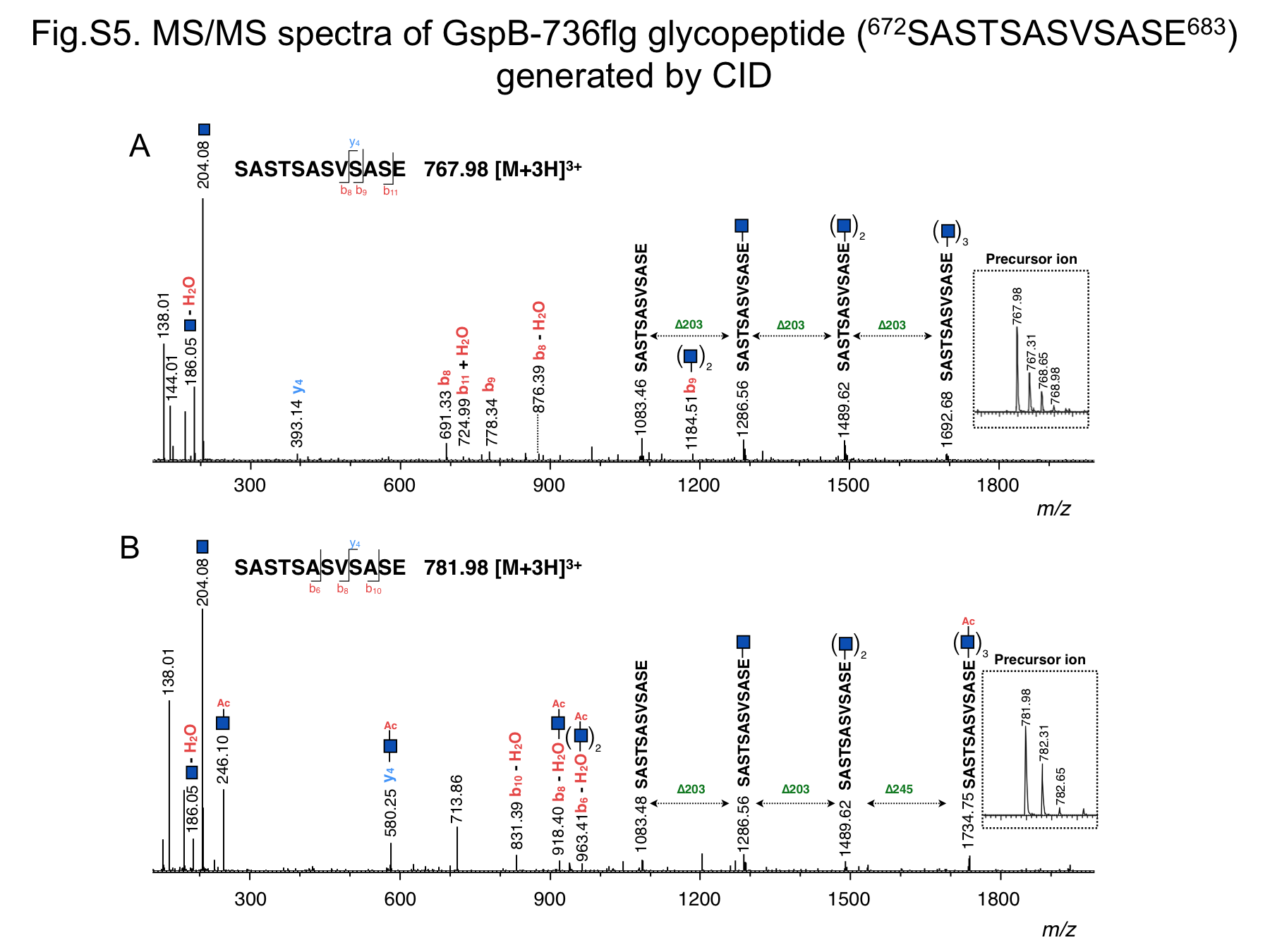

Supplement: S5 Fig — (A) Fragmentation spectrum of peak 3, m/z = 767.98 [M+3H]3+ O-glycosylated at six positions. (B) Fragmentation spectrum of peak 3*, m/z = 781.98 [M+3H]3+ O-glycosylated at six positions and O-acetylated at one position. O-GlcNAc modifications are designated by a blue square. MS spectra of precursor ions are shown in the insets. (TIFF) [file ppat.1006558.s005.tiff]

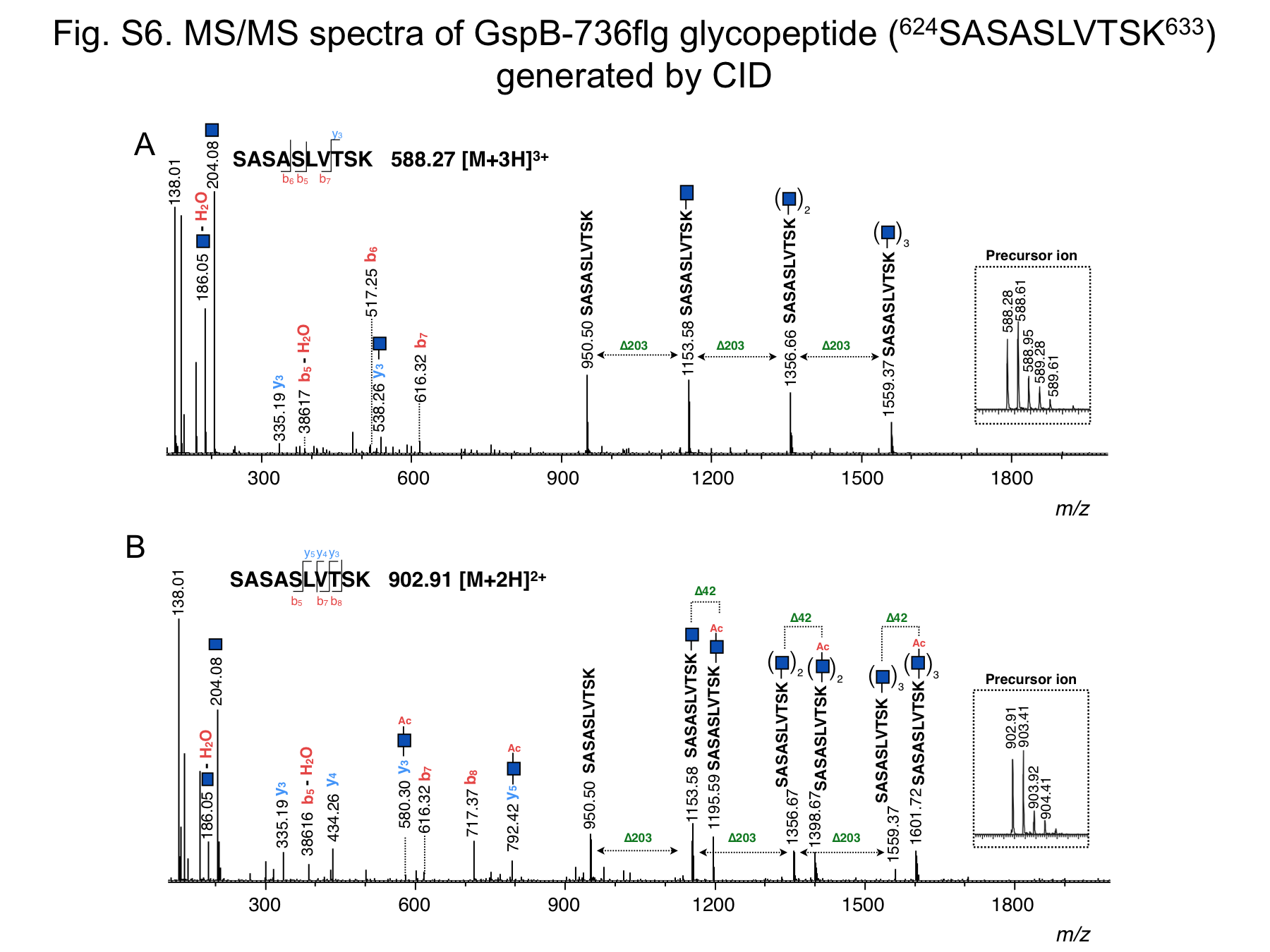

Supplement: S6 Fig — (A) Fragmentation spectrum of peak 4, m/z = 588.27 [M+3H]3+ O-glycosylated at four positions. (B) Fragmentation spectrum of peak 4*, m/z = 902.91 [M+2H]2+ O-glycosylated at four positions and O-acetylated at one position. O-acetyl modifications are designated by a blue square. MS spectra of precursor ions are shown in the insets. (TIFF) [file ppat.1006558.s006.tiff]

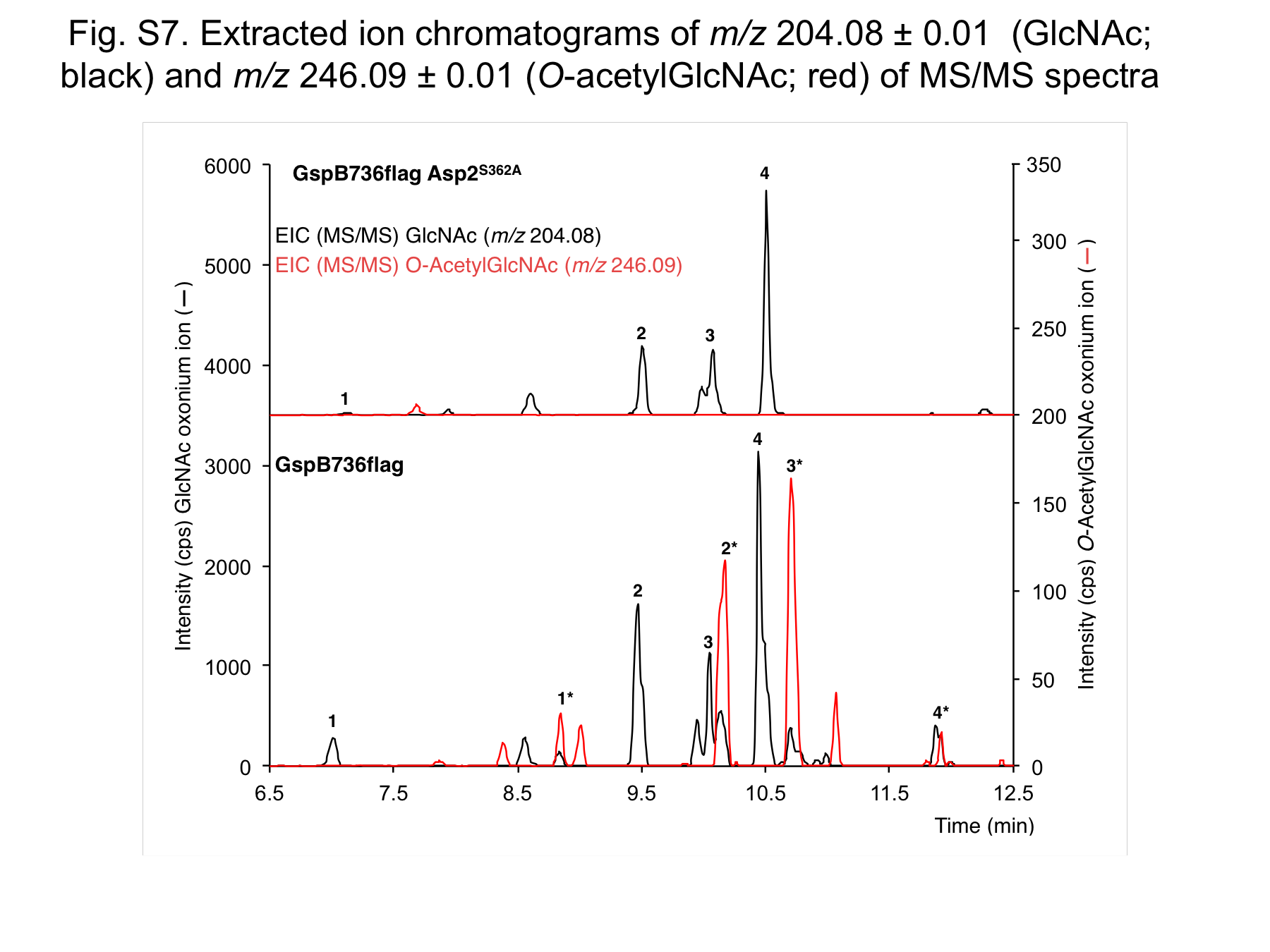

Supplement: S7 Fig — (TIFF) [file ppat.1006558.s007.tiff]

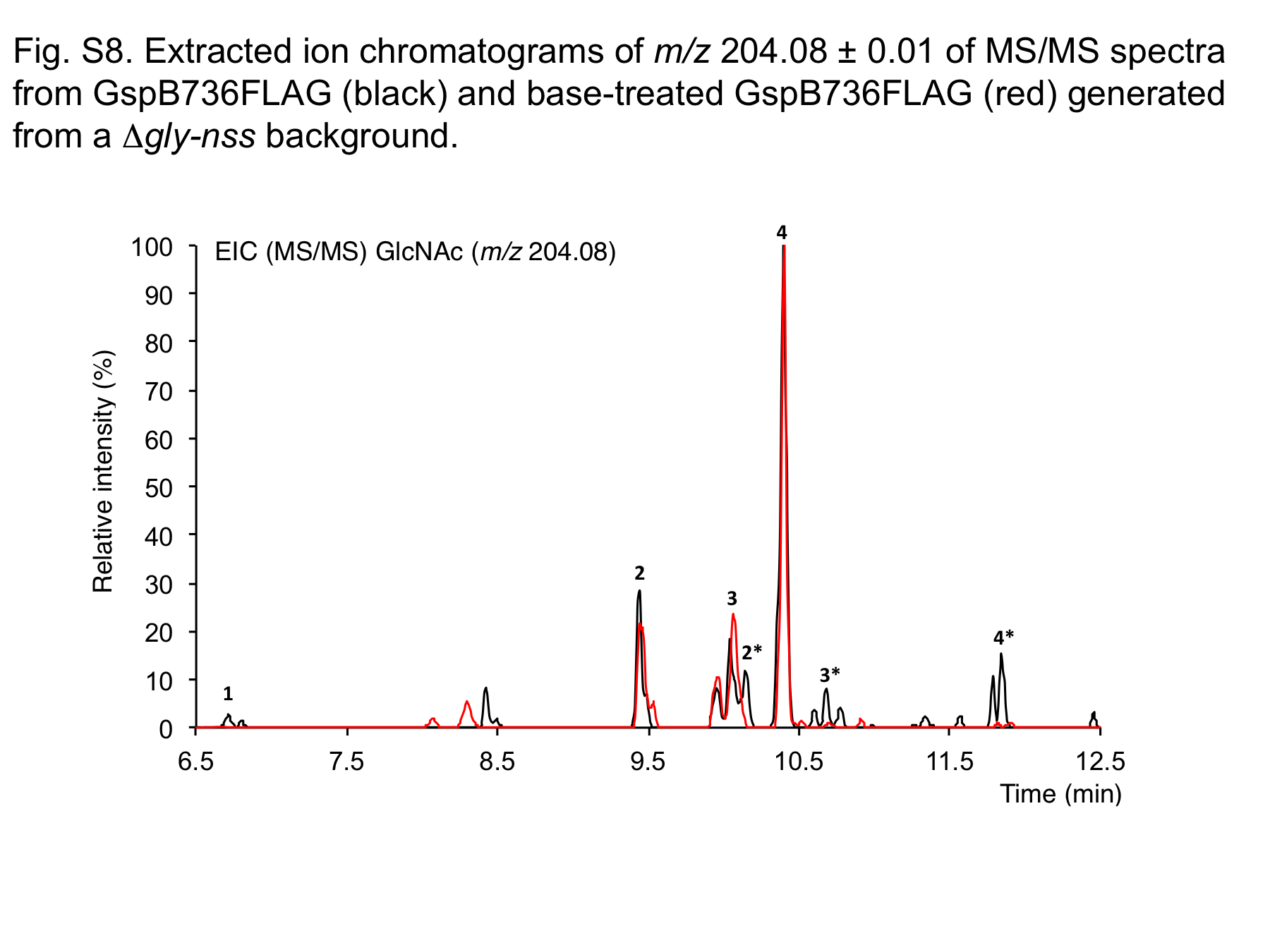

Supplement: S8 Fig — (TIFF) [file ppat.1006558.s008.tiff]

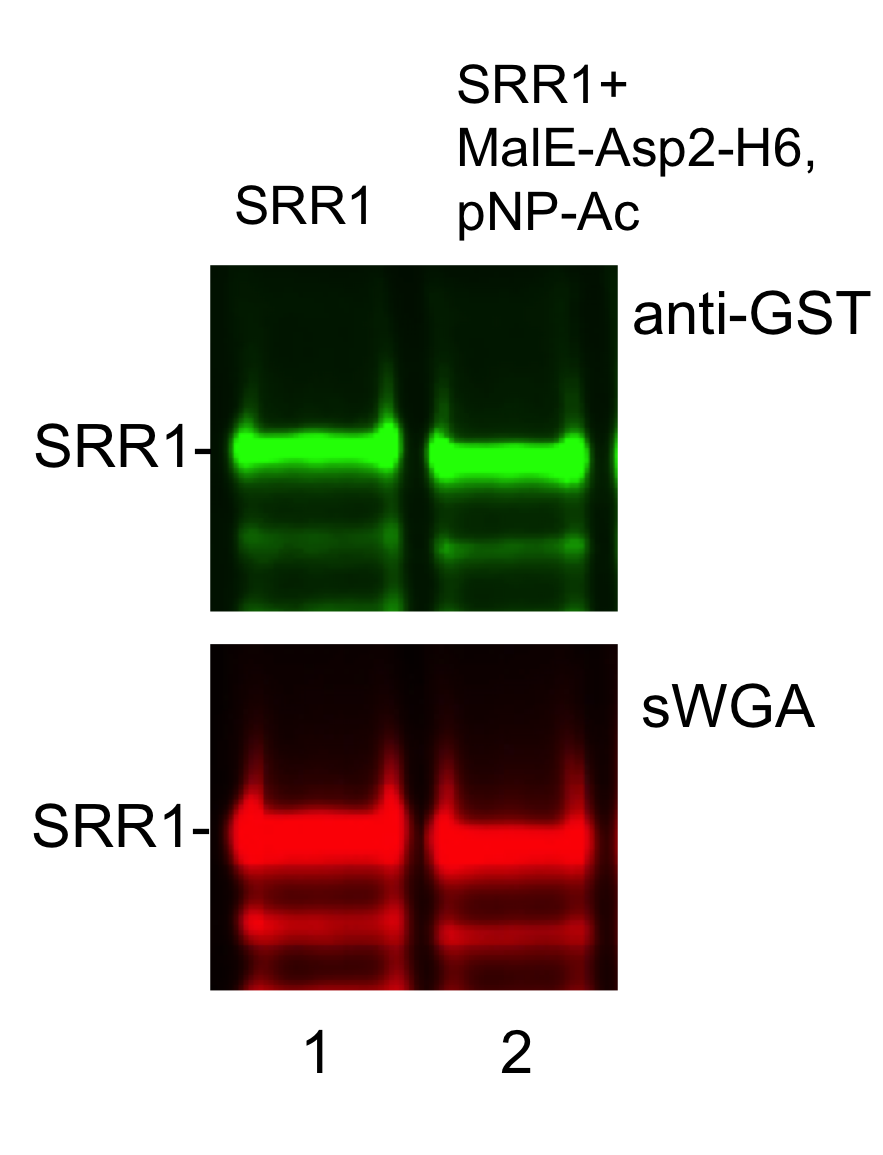

Supplement: S9 Fig — Western blot analysis of glycosylated SRR1 (lane 1), or glycosylated SRR1 co-incubated with pNP-Ac and MalE-Asp2-H6 (lane 2) (as described in methods and materials). O-acetylation in vitro reactions were stopped after 1 hr incubation by mixing with protein sample buffer (Novagen). Proteins were separated by SDS-PAGE and subjected to Western blot analysis using anti-GST antibodies or lectin blot analysis using biotinylated sWGA to determine glycosylated SRR1 levels and GlcNAc reactivity, respectively. (TIFF) [file ppat.1006558.s009.tiff]

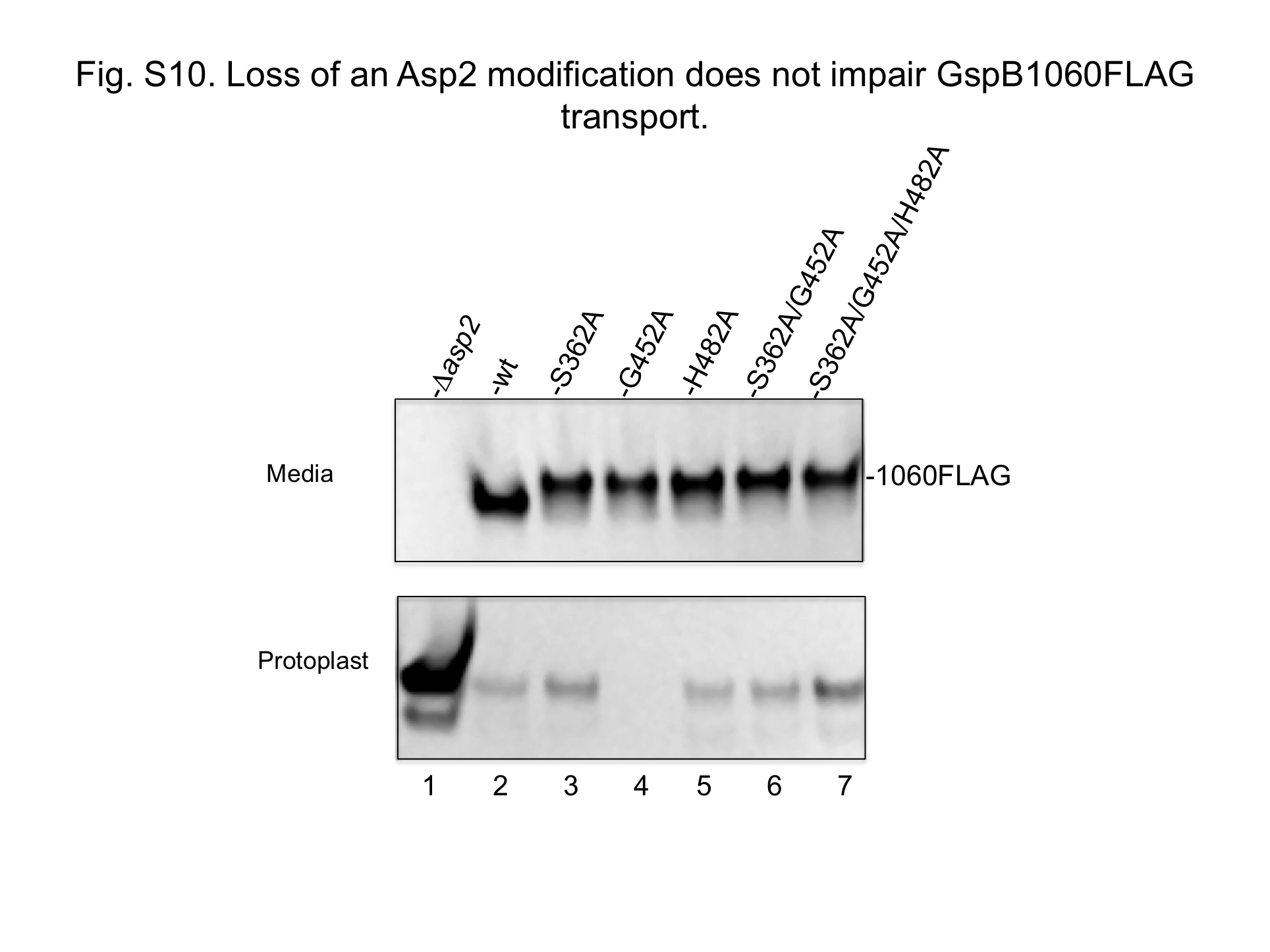

Supplement: S10 Fig — Western blot analysis of Asp2-dependent export of GspB1060flag by S. gordonii Δasp2 strain PS3349 (lane 1), parental strain PS921 (lane 2) and derivative strains harboring the designated alanine substitution within the catalytic triad of Asp2, PS3541 (lane 3), PS3555 (lanes 4), PS3556 (lane 5), PS3557 (lane 6), PS3558 (lane 7). Culture media was collected from exponentially growing strains. Proteins were separated by SDS-PAGE (3–8%) and analyzed by Western blotting, using anti-FLAG antibody to detect GspB736flag. (TIF) [file ppat.1006558.s010.tif]

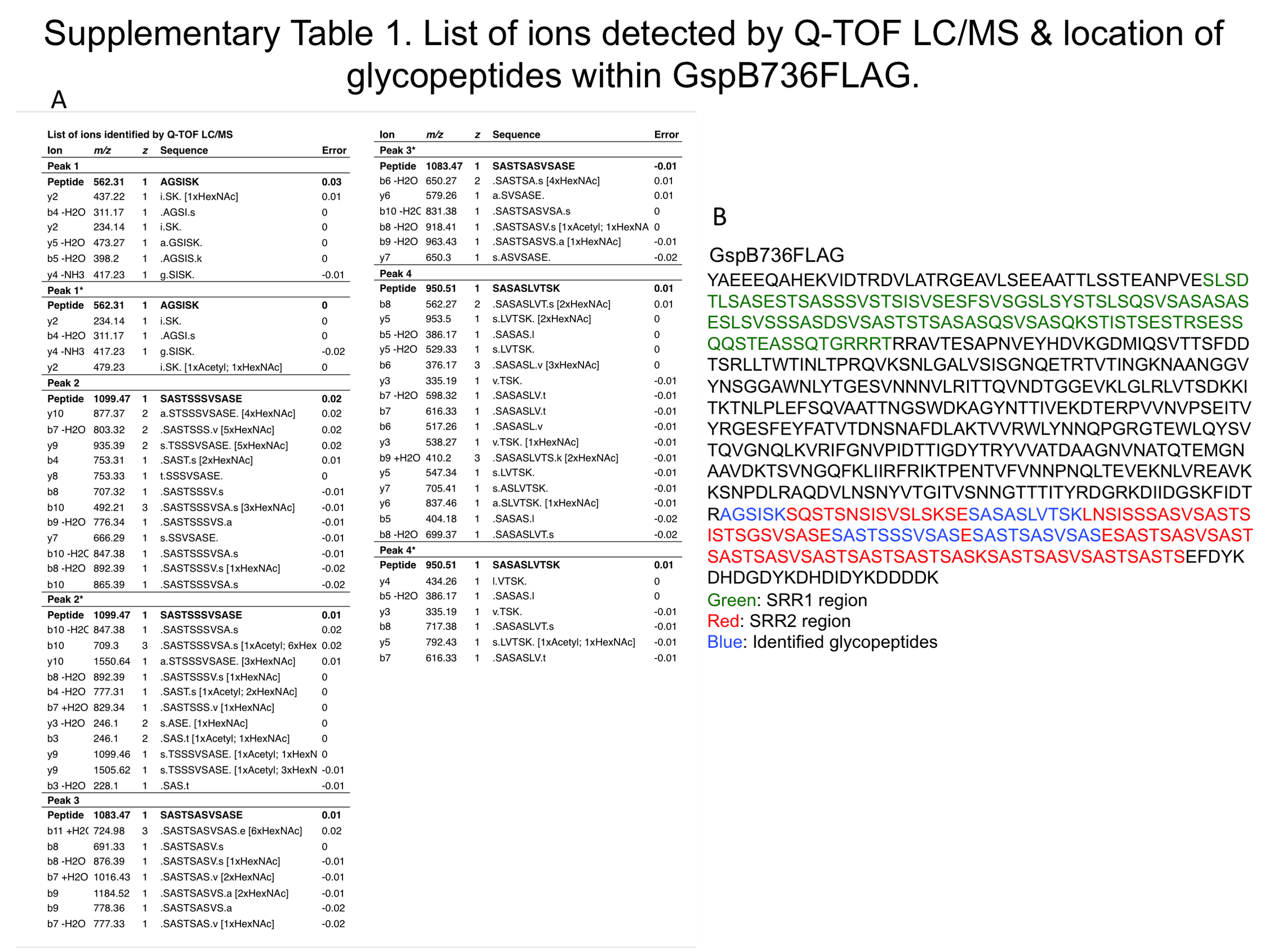

Supplement: S1 Table — (A) List of ions identified by Q-TOFF LC/MS from each of the four major glycopeptide fragments generated from GspB736FLAG protease digestion (Peaks 1–4). (B) Glycopeptide fragment location within the GspB736FLAG amino acid sequence. The first and second glycosylated serine rich repeat regions (SRR1 and SRR2) within GspB are shown in green and red respectively. Glycopeptide fragments corresponding to peaks 1–4 are highlighted in blue. (TIF) [file ppat.1006558.s011.tif]
